# X-ray stimulates NQO1-dependent cascade reactions to induce strong immunogenicity for MRI-guided cancer radio-chemodynamic-immunotherapy

**DOI:** 10.7150/thno.110573

**Published:** 2025-06-09

**Authors:** Li He, Jiao-Jiao Ma, Yi-Qun Wu, Chen-Guang Wang, Tong Lan, Lu Su, Lin Zhu, Shi-Wen Huang, Kai Deng, Yong-Chang Wei

**Affiliations:** 1 Department of Radiation and Medical Oncology, Zhongnan Hospital of Wuhan University, Wuhan 430071, China.; 2 Department of Radiology, Zhongnan Hospital of Wuhan University, Wuhan 430071, China.; 3 Key Laboratory of Biomedical Polymers of Ministry of Education, Department of Chemistry, Wuhan University, Wuhan 430072, China.; 4 Department of Orthopedic Trauma and Microsurgery, Zhongnan Hospital of Wuhan University, Wuhan 430071, China.; 5 Hubei Bio-Pharmaceutical Industrial Technological Institute Inc., Wuhan 430205, China.

**Keywords:** NQO1, immunogenicity, hydroxyl radicals, radioimmunotherapy, chemodynamic therapy

## Abstract

**Background:** Immunogenicity activation is vital for radioimmunotherapy, but the short-term oxidative damage caused by precise radiation planning limits this effect. Chemodynamic therapy (CDT) with prolonged generation of hydroxyl radical (•OH) can initiate immunogenicity in combination with X-rays, however, its performance is constrained by tumor insufficient H2O2.

**Methods:** Here, we propose to construct β-lapachone-based nanoparticles (β-Lap/Fe NPs) which initiate cascade reactions to generate high levels •OH for an extended period in tumor following X-ray irradiation.

**Results:** β-Lap/Fe NPs, constructed by co-encapsulation of β-Lap and Fe3O4 nanoparticles in reactive oxygen species (ROS) responsive C16-S-mPEG2000 micelles, remain stable under normal conditions but rapid decompose and release β-Lap and Fe2+ when exposed to high level ROS. Upon X-ray irradiation, the upregulation of ROS and NAD (P) H: quinone oxidoreductase-1 (NQO1) in tumor cells accurately triggers β-Lap/Fe NPs to persistently generate high levels H2O2 and •OH for 12 hours, ultimately causing strong immunogenic cell death effects. Moreover, β-Lap/Fe NPs with excellent T2-weighted magnetic resonance imaging provide imaging reference for guiding precise X-ray radiation and predicting •OH generation. β-Lap/Fe NPs mediated radio-chemodynamic-immunotherapy remarkably against primary tumor growth, and further shows effective suppression on untreated distant tumors via the abscopal effect.

**Conclusions:** In a word, this work proposed the simple but powerful strategy for cancer radio-chemodynamic-immunotherapy that combines X-ray and CDT to remote locally and visually actuated long-time production of H2O2 and subsequently persistent generation of •OH for initiating strong antitumor immune responses.

## Introduction

Breast cancer is the most common malignant tumor and the leading cause of cancer-related deaths among women globally. It is the second leading cause of global cancer incidence in 2022, accounting for approximately 11.6% of all new cancer cases. The conventional treatment approaches of breast cancer include surgical resection, chemotherapy, radiotherapy (RT) and targeted therapy. Radiotherapy plays an important role in the treatment of breast cancer, especially in breast-conserving surgery and the treatment of locally advanced breast cancer. Radiotherapy is able to provide curative or palliative treatment for up to 50% of cancer patients, with less systemic toxicity than conventional chemotherapy 1-3. Theoretically, high-energy ionizing radiation, including X-rays, locally induces cancer cell death through direct DNA damage and indirect reactive oxygen species (ROS) generation 4,5. In addition to combating primary tumors, RT can potentially initiate innate and adaptive immune responses to suppress tumor metastasis 6,7. Typically, upon ionizing radiation, the generation of ROS, especially the most reactive and toxic hydroxyl radical (•OH), can induce immunogenic cell death (ICD), characterized by the release of tumor-associated antigens (TAAs) and damage-associated molecular patterns (DAMPs) from dying cancer cells 8,9. The dendritic cells (DCs) engulf these released or exposed DAMPs and TAAs, then process and present them to naive T cells, initiating maturation and infiltration of cytotoxic T cells in the tumor site. Considering the overexpressed PD-L1 on the surface of cancer cells stimulated by RT exhausts the tumoricidal effect of cytotoxic T cells, the combination of RT and immune checkpoint blockade (ICB) therapy has been extensively explored in preclinic and clinic 10,11. However, this radioimmunotherapy shows unsatisfactory performance in preclinic and clinic.

Numerous reports suggest that the therapeutic efficiency of radioimmunotherapy is dramatically restricted by X-ray-induced weak immunogenicity of tumor cells, which is mostly attributed to inadequate oxidative damage upon X-ray radiation. To elevate intracellular oxidative stress, many radiosensitizers, including metal-based nanomaterials with high-Z elements, have been applied to accelerate water radiolysis by absorbing and depositing ionizing radiation, temporarily elevating the cellular oxidative stress through the generation of •OH 12-15. Although these radiosensitizers reinforce X-ray-caused oxidative damage and make certain progress in combating cancer, they are insufficient to meet clinical requirements. Due to the antioxidant mechanism of cancer cells and the highly reactive and short lifespan of •OH (<40 ns), it is challenging to maintain oxidative stress in cancer cells for an extended period under precise X-ray dose planning, which provides cancer cells the opportunity to recover from radiation damage. The strategies to inhibit or destroy antioxidant mechanism of cancer cells are helpful for reinforced oxidative stress in radiotherapy 16-18. However, cancer cells, which possess robust antioxidant capabilities, can initiate alternative antioxidant pathways or compensatory antioxidant mechanisms, thereby attenuating oxidative damage and even inducing resistance to radiotherapy. Considering the difficulty in prolonging the lifetime of •OH, a strategy that extends the duration of high-level •OH generation may reinforce the oxidative damage following radiotherapy.

Chemodynamic therapy (CDT), which utilizes Fenton or Fenton-like reactions to catalyze lowly toxic H_2_O_2_ to produce highly lethal •OH, may aid in sustaining the oxidative damage in cancer cells when combined with X-ray irradiation 19-24. For one thing, CDT has the advantages of no drug resistance and minimal adverse effects. For another, X-ray irradiation can positively regulate Fenton or Fenton-like reactions by inducing the acceleration of mitochondrial respiration to increase the production of H_2_O_2_. Furthermore, in the presence of H_2_O_2_, the generation of •OH mediated by Fenton or Fenton-like reactions can continue indefinitely, which is beneficial for maintaining high levels of oxidative stress within cancer cells after X-ray irradiation. However, the level of H_2_O_2_ directly upregulated by ionizing radiation is insufficient for Fenton or Fenton-like reactions 25. Although the delivery of glucose oxidase, metal peroxides, exogenous H_2_O_2_, etc. can upregulate the intratumor H_2_O_2_ and accelerate Fenton or Fenton-like reactions, these methods lack specificity and carry the risk of generation of H_2_O_2_ in normal tissues 26-30. Therefore, developing a strategy to fix-point reinforced the level of H_2_O_2_ in cancer cells is efficient and safe for the combination of radiotherapy and CDT. Nicotinamide adenine dinucleotide (phosphate) (NAD(P)H): quinone oxidoreductase1 (NQO1), a cytosolic two-electron oxidoreductase that catalyzes quinones to the corresponding hydroquinones using endogenous hydride source, NAD(P)H, has been a promising target for improving Fenton chemistry 31-33. Typically, β-lapachone possesses a unique quinone structure and can be converted by NQO1 through a futile redox cycle to continuously generate large amounts of H_2_O_2_ and disturb NAD(P)H mediated antioxidative mechanism 34-37. The lethality of β-Lap against tumor cells is NQO1-dependent, positively correlated with the levels of NQO1. Although, the level of NQO1 in cancer cells is heterogeneous, it can be significantly upregulated in cancer cells upon X-ray irradiation 38-39. Meanwhile, it has been demonstrated that the combination of β-Lap with mild hyperthermia significantly enhances cytotoxic effects in lung cancer cells, suggesting a potentiated antitumor response compared to either treatment alone. Additionally, research by Lamberti et al. revealed a pronounced synergistic effect when β-Lap was used in conjunction with photodynamic therapy, indicating that β-Lap holds considerable promise in combination treatment regimens to improve therapeutic efficacy against tumors 40-41. Accordingly, we hypothesize that β-Lap can potentially mediate efficient Fenton reactions and long-term oxidative damage through precise and prolonged elevation of intracellular H₂O₂ levels after X-ray irradiation, ultimately initiating potent antitumor immunogenic effects.

**Scheme 1:**
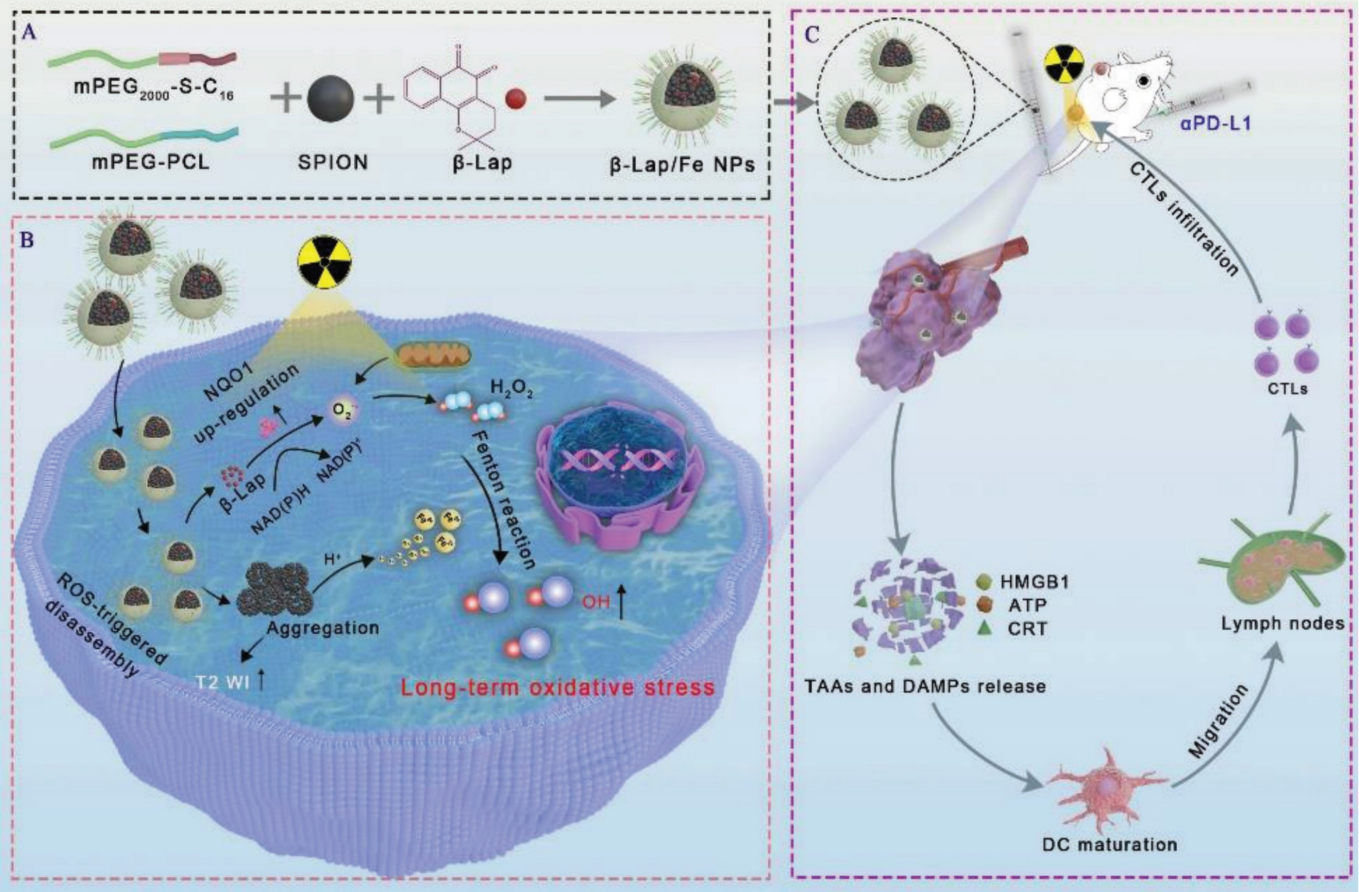
Schematic illustration of β-lapachone-based nanoparticles (β-Lap/Fe NPs) for sustained ·OH generation under X-ray irradiation to induce vigorous immunogenicity for radio-chemodynamic-immunotherapy (RCDI). A) The co-encapsulation of Fe3O4 NPs and β-Lap with ROS-responsive polymers (C16-S-mPEG2000) and mPEG-PCL (Mw = 5000). B) The mechanism of β-Lap/Fe NPs stimulating potent immunogenic effects upon X-ray irradiation: X-ray upregulated ROS and NQO1 in tumor cells trigger cascade reactions with β-Lap/Fe NPs for amplified oxidative stress by persistently generating high-levels ·OH. C) β-Lap/Fe NPs initiate strong immune response upon X-ray irradiation for mediating efficient cancer RCDI.

In this work, we developed β-lapachone-based nanoparticles (β-Lap/Fe NPs) to mediate strong immunogenicity by locally persistent generation of **·**OH after X-ray irradiation for cancer radio-chemodynamic-immunotherapy (RCDI). As depicted in Scheme 1, β-Lap/Fe NPs were prepared from the co-encapsulation of β-lapachone and Fe_3_O_4_ nanoparticles in ROS-responsive amphiphilic polymers (mPEG_2000_-S-C_16_). β-Lap/Fe NPs were stable in a normal physiological environment, while destroyed under high-level ROS, releasing β-lapachone and Fe^2+^. *In vitro*, β-Lap/Fe NPs were difficult to collapse without X-ray irradiation, while could accessibly be destroyed and release β-lapachone and Fe^2+^ by X-ray induced elevation of ROS. Meanwhile, the elevation of cellular NQO1 triggered by X-ray catalyzed the released β-lapachone to continuously consumed NAD(P)H and generated higher levels of H_2_O_2_. This process promoted the sustained generation of **·**OH for 12 hours in the presence of Fe^2+^, resulting in long-term oxidative damage and strong immunogenic cell death (ICD) effects. *In vivo*, β-Lap/Fe NPs exhibited enhanced T2-weighted magnetic resonance imaging (T2 WI) in tumors with improved permeability and retention (EPR) effect, precisely guiding X-ray irradiation. Upon X-ray radiation, the elevation of ROS in the tumor led to the sequential collapse of β-Lap/Fe NPs and aggregation of Fe_3_O_4_ nanoparticles, further enhancing the T2 WI, predicting **·**OH production and oxidative stress enhancement. As a result, the persistent generation of **·**OH triggered tumor cell death and strong ICD effects, significantly inhibiting the growth of primary tumors and untreated distant tumors when combined with αPD-L1. In a word, this work proposed the simple but powerful strategy for cancer radio-chemodynamic-immunotherapy that combines X-ray and CDT to remote locally and visually actuated long-time production of H_2_O_2_ and subsequently persistent generation of **·**OH for initiating strong antitumor immune responses.

## Materials and Methods

### Materials

1-hexadecanol (C_16_-OH), ‌thiodiglycolic anhydride, N,N′-dicyclohexylcarbodiimide (DCC), 4-dimethylaminopyridine (DMAP), and iron acetylacetone (Fe(acac)_3_) were obtained from Aladdin Reagent Co. (Shanghai, China). Polyethylene glycol monomethyl ether (mPEG, Mn = 2000) was purchased from TCI Development Co., Ltd (Shanghai, China). β-Lapachone (β-Lap) was purchased from Qiyue Biotechnology Co., Ltd (Xian, China). All of the organic solvent including dichloromethane (DCM), trichloromethane, N,N-Dimethylformamide (DMF), dimethyl sulfoxide (DMSO), diphenyl oxide, oleylamine, ethyl ether and ethyl alcohol were purchased from Sinopharm Chemical Reagent Co., Ltd (Shanghai, China). Biological reagents such as 4',6'-diamidino-2-phenylindole (DAPI), Hoechst 33342, 3-(4,5-Dimethyl-2-Thiazolyl)-2,5-Diphenyl Tetrazolium Bromide (MTT) and 2,7-dichlorodihydrofluorescein diacetate (DCFH-DA) were obtained from Beyotime Biotechnology Co., Ltd (Shanghai, China). Prussian Blue Iron Stain Kit was obtained from Solarbio Science & Technology Co., Ltd (Beijing, China). Hydrogen peroxide assay kit and Nanjing Jiancheng Bioengineering Institute (Nanjing, China). All the antibodies were purchased from Dakewe Biotech Co., Ltd (Beijing, China).

### Cells and animals

4T1 cell line were obtained from Procell Life Science & Technology Co., Ltd (Wuhan, China). 4T1 cells were cultured in RPMI 1640 medium supplemented with 10% fetal bovine serum and 1% penicillin and streptomycin. The cells were cultured at 37 °C in a humidified atmosphere of 5% carbon dioxide (CO_2_).

Animal procedures were performed using 6-8 weeks old Balb/c female mice. All animals used in the study were purchased from Changsheng Biotechnology Co., Ltd (Liaoning, China). Animal ethics approval came from the Experimental Animal Welfare Ethics Committee, Zhongnan Hospital of Wuhan University (ZN2024089).

### Synthesis of mPEG_2000_-S-C_16_

Firstly, 1-hexadecanol (2.42 g, 10 mmol) and thioglycolic anhydride (1.98g, 15 mmol) were added into a 100 mL flask with a mixture solvent consisting of dry DMF (5 mL) and dry DCM (30 mL) and then stirred for 6 h at room temperature under N_2_ atmosphere. After the reaction, the crude product was purified by column chromatography using dichloromethane/methanol (v/v, 20:1) as eluent. C_16_-S-COOH was obtained as faint yellow solid. Subsequently, mPEG_2000_ (1 equiv), C_16_-S-COOH (1.5 equiv), DCC (1.5 equiv), DMAP (1.5 equiv) and 10 mL dry DCM were charged into 25 mL flask and stirred at room temperature for 48 h under N_2_ atmosphere. After removing the precipitation, the collected filtrate was enriched by evaporation by rotary evaporator and then dropped into the ice ethyl ether for sedimentation. Then the settling was collected and dissolved in few DCM after which the mixture was settled in ice ethyl ether. After repeated three times, mPEG_2000_-S-C_16_ was obtained by filtration and vacuum drying.

### Preparation of superparamagnetic iron oxide nanoparticles (Fe_3_O_4_ NPs)

The Fe_3_O_4_ nanoparticles were synthesized via a thermal decomposition procedure referring to previous studies. Briefly, diphenyl oxide (10 mL) and oleylamine (10 mL) were poured into a 100 mL flask as the reaction solvent, subsequently, Fe(acac)_3_ (0.7 g, 2 mmol) was added into the flask and the reaction system was heated from room temperature to 110 °C for 1 h under nitrogen atmosphere. The mixture was then rapidly heated to 300 °C, maintaining at this temperature for 2 h. The mixture was cooled naturally and ethanol was added into it. The Fe_3_O_4_ nanoparticles were separated by centrifugation at 8000 r/min for 10 min. The obtained Fe_3_O_4_ nanoparticles were washed with ethanol three times, and finally suspended with trichloromethane and preserved at 4 °C away from light for further use.

### Preparation of β-Lap/Fe NPs

The mPEG_2000_-S-C_16_ (50 mg), mPEG-PCL (10 mg), β-Lap (5 mg), and Fe_3_O_4_ NPs (5 mg) were dissolved in trichloromethane/THF (v/v, 2/1, 3 mL) and then deionized water (20 mL) was added into it, followed by emulsifying by sonication for 15 min under the ice bath. The β-Lap/Fe NPs were obtained by evaporation to remove organic solvent and centrifuging to remove uncoated drugs. The Fe NPs were prepared in the same way without adding β-Lap. The β-Lap NPs were prepared by the flash nano-precipitation method. Briefly, mPEG_2000_-S-C_16_ (50 mg), mPEG-PCL (10 mg) and β-Lap (5 mg) were dissolved in THF (1 mL) and quickly added into deionized water (9 mL) under vigorous stirring. Then the mixture was placed into dialysis bag (MWCO, 3500 Da) and removed organic solvent by dialysis against deionized water for 24 h, and the obtained β-Lap NPs were kept at 4 °C.

### ROS-sensitive of β-Lap/Fe NPs

The ROS-sensitive of C_16_-S-mPEG_2000_ polymer was studied by ^1^H NMR. C_16_-S-mPEG_2000_ (200 mg) was dissolved in 10 mL deionized water. Then the solution was evenly mixed with H_2_O_2_ solution (1 M, 10 mL) so that the final concentration of H_2_O_2_ in the mixed solution was 1 mM. Subsequently, the mixed solution was incubated on a shaking bed at 37 °C. At the predetermined time interval, 1 mL reaction solution was taken out and lyophilized. The dried product was dissolved in CDCl_3_ for ^1^H NMR.

β-Lap/Fe NPs solution (10 mg/mL) was respectively reacted with PBS or H_2_O_2_ solution (0.1 mM or 1 mM) at 37 °C for different times to study the variation of micelle size. At the predetermined time interval, the micellar sizes were measured by DLS and the morphologies of the micelles were studied by TEM.

### ROS-triggered β-lapachone release

5 mL of C_16_-S-mPEG_2000_ micelles solution was mixed with 5 mL H_2_O_2_ solution (2 mM or 0.2 mM). Subsequently, the reaction solution was transferred into the dialysis bag (MWCO, 3500 Da), the dialysis bag was then placed into a tube containing 30 mL PBS (pH 7.4) and incubated at 37 °C. At the determined time interval, 3 mL of the solution was taken out, followed by appending 3 mL of PBS and keeping shaking. The concentration of β-Lap was detected by UV-VIS spectrophotometer at 257 nm and the cumulative drug release was calculated. The experiment was repeated three times.

### The Fenton reaction

The efficiency of the Fenton reaction was evaluated indirectly by methylene blue (MB). Hydroxyl radicals (•OH) produced by Fenton reaction can contribute to degradation of MB. The β-Lap/Fe NPs solution was prepared, H_2_O_2_ solution (final con-centration 1 mM) and MB solution (final concentration 5 µg/mL) were then added. After incubating at 37 °C for 1 h, the absorbance was measured by UV-VIS spectrophotometer.

### *In vitro* cytotoxicity

To evaluate the cytotoxicity, 4T1 cells (3×10^3^ cells/well) were seeded into 96-well cell culture plates and incubated for 24 h. The cells were treated with a range of concentrations of nanoparticles for 2 h and then replaced with fresh medium and exposed to 2 Gy irradiation or sham irradiation. The cells were further incubated for 24 h. Then, MTT (20 μL, 5 mg/mL) was added and incubated at 37 °C for 2 h. Subsequently, all the medium was replaced with DMSO (150 μL) to dissolve the obtained crystals and then the absorbance of each well at 570 nm was detected by a microplate reader. Then cell viability was calculated as follows (n = 3): Cell viability (%) = (A_sample_ - A_0_)/(A_control_ - A_0_) × 100%, where A represents the absorbance at 570 nm.

### *In vitro* cell apoptosis assay

4T1 cells (2 × 10^5^ cells/well) were seeded into 6-well cell culture plates and incubated for 24 h. β-Lap/Fe NPs (β-Lap = 3 μM) were added and incubated for 2 h. The cells were then treated with or without 2 Gy irradiation after the replacement of fresh medium. And after incubation for another 24 h, the cells were collected and stained with incubated with Annexin V-FITC/PI apoptosis detection kit. The stained cells were analyzed by flow cytometry immediately.

### Live-dead cells staining

4T1 cells were seeded in 12-well cell culture plates with a density of 1×10^5^ cells per well. After incubation for 24 h, the cells were treated with β-Lap/Fe NPs (β-Lap = 3 μM). Then the medium was replaced 2 h later and the cells were exposed with or without 2 Gy irradiation, immediately. The cells were incubated for another 24 h and then the cells were stained with Calcein-AM and PI for 30 min. The image was observed under inverted fluorescence microscope (IFM).

### Intracellular DNA damage detection

4T1 cells were seeded into confocal culture dishes with a density of 2×10^5^ cells per well. After incubating overnight, the cells were treated with medium containing β-Lap/Fe NPs. Fresh medium was replaced after 2 h incubation and 2 Gy irradiation was given immediately. After X-ray irradiation, the cells were continued to be cultured and then fixed with 4 % paraformaldehyde at the predetermined time (6 h or 12 h), rinsed with PBS for three times. Subsequently, the cells were permeated at room temperature with 0.3% TritonX-100 for 10 min, and then blocked with PBS containing 0.1 % Tween 20 and 5% BSA for 1 h. After discarding the solution, the cells were incubated with anti-γ-H_2_AX (1:500) antibody overnight at 4 °C. After rinsed with PBS, the cells were incubated with secondary antibody (anti-rabbit IgG 1:1000) conjugated with FITC at room temperature. The cells were incubated for 1 h in the dark and washed by PBS for three times, subsequently stained with DAPI. After rinsed with PBS, the cells were observed by Confocal laser scanning microscope (CLSM) instantly.

### Detection of ICD biomarkers *in vitro*

Calreticulin (CRT) exposure analysis: FCM and CLSM were used for the analysis of CRT exposure. 4T1 cells (2 × 10^5^ per well) were seeded into 6-well cell culture plates or confocal culture dishes and incubated for 24 h. Then the cells were treated with β-Lap/Fe NPs respectively. After incubation for 2 h, fresh medium was replaced and the cells were irradiated with 2 Gy. Subsequently, the cells were incubation for additional 24 h. Afterward, the cells seeded in the 6-well cell culture plates were used for FCM, briefly, the cells were collected and incubated with anti-CRT antibody (1:1000) conjugated with FITC for 30 min, washed and analyzed by FCM. On the other hand, the cells seeded in the confocal culture dishes were used for CLSM. The cells were incubated with anti-CRT antibody (1:200) conjugated with FITC for 30 min, after which washed with PBS and fixed with 4 % paraformaldehyde. And then the cells were stained with DAPI, rinsed with PBS, and observed by CLSM.

High mobility group box-1 protein (HMGB1) release analysis: The HMGB1 was detected by CLSM. 4T1 cells were seeded into confocal culture dishes and then treated with the same way as above. After incubation for 24 h, the cells were fixed with 4% paraformaldehyde and then 0.3% TritonX-100 was permeated at room temperature for 10 min, and then closed with 5% BSA solution for 1 h to reduce non-specific binding. HMGB1 (1:500) was incubated overnight at 4 °C, and after rinsing with PBS, Cy5-labeled fluorescent secondary antibody (1:2000) was added and incubated at room temperature for 1 h away from light. DAPI was used for staining the nucleus. The distribution of HMGB1 was observed by CLSM.

Adenosine triphosphate (ATP) release analysis: 4T1 cells were seeded into 6-well cell culture plates and treated with the same way as above. The supernatant was collected and detected by ATP assay kit for ATP release analysis.

### Intracellular ROS monitoring

4T1 cells (1 × 10^5^ per well) were seeded into 12-well cell culture plates. After attachment overnight, the cells were exposed to 2 Gy irradiation or sham irradiation after incubation with medium containing β-Lap/Fe NPs for 2 h. The cells were then incubated in the incubator for another 6 h or 12 h, after that the cells were stained with a ROS probe DCFH-DA (10 μM) or a •OH probe BBoxiProbe®O28 (10 μM) at 37 °C for 30 min. After staining, all the medium was removed and the cells were imaged under an inverted fluorescence microscope (IFM) after washing.

### *In vitro* magnetic resonance imaging

The β-Lap/Fe NPs and N-β-Lap/Fe NPs were configured into 200 μg/mL, 100 μg/mL, 50 μg/mL, 25 μg/mL, 12.5 μg/mL, and 6.25 μg/mL aqueous solutions, and then added into the 96-well plate and imaged on the 3.0 T MR scanner. The imaging parameters are as follows: TR = 3810 ms, TE = 66 ms.

### *In vivo* magnetic resonance imaging

T2 MRI imaging was performed in 4T1 tumor-bearing mice. The mice were anesthetized and imaged with 3.0 T MR scanner to obtain the image data before administration. Then the mice were injected intravenously with β-Lap/Fe NPs or N-β-Lap/Fe NPs ([β-Lap] = 0.1 mg/kg) and scanned at a predetermined time point. The imaging parameters are as follows: TR = 4000 ms, TE = 67 ms.

### *In vivo* antitumor efficacy

For establishment of subcutaneous tumor model, 4T1 cells (1 × 10^6^) were subcutaneously injected into the right flank of each mice. When the tumor size reached 150 mm^3^, the mice were randomly divided into six groups (n = 5 in each group) and each group correspondingly were treated with PBS, β-Lap/Fe NPs, RT, RT + Fe NPs, RT + β-Lap NPs, and RT + β-Lap/Fe NPs ([β-Lap] = 0.1 mg/kg). The mice were injected intravenously with PBS or NPs through a tail vein, and exposed to 2 Gy irradiation or sham irradiation 2 h later. The treatment was repeated every other day for a total of 5 times. The length and width of the tumor and the body weights of the mice were measured and recorded every other day. The tumor volume was estimated using the formula: Volume = 0.5×Length^2^×Width^2^. On day 24, the mice were sacrificed and the tumor tissues of the mice were collected and fixed with 4% paraformaldehyde for later H&E staining, TUNEL staining and Ki67 staining.

### The immune evaluation

The mice were sacrificed to collect the tumor tissues and tumor-draining lymph nodes (TDLNs) on day 11. The tumor tissues were digested into single cells by incubating with 1640 medium containing collagenase IV, DNase I and hyaluronidase at 37 °C for 1 h. The collected single tumor cells were incubated with medium containing Leukocyte Activation Cocktail with Golgiplug (BD Pharmingen) for 4-6 h in a cell culture incubator. Then the tumor cells were stained with anti-CD45-APC-CY7, anti-CD3-FITC, anti-CD4-PE, anti-CD8-APC, anti-IFN-γ-PE-CY7 and anti-Foxp3-BV421 for T cells analysis. TDLNs were prepared into single cells by grinding flush repeatedly. Then the cells from TDLNs were stained with anti-CD11c-FITC, anti-CD80-PE and an-ti-CD86-APC to evaluate DCs maturation.

### Systemic antitumor performance

A bilateral subcutaneous tumor model was established to evaluate systemic immune activation. 4T1 cells (1 × 10^6^) were subcutaneously injected into the right flank on day 7 to establish the primary tumors. Three days later, 4T1 cells (2 × 10^5^) were subcutaneously injected into the left flank to establish the distant tumors. When the primary tumors reached 150 mm^3^ in volume, tumor bearing mice were randomly divided into 5 groups (n = 7 in each group): PBS, β-Lap NPs, RT, αPD-L1, RT + αPD-L1, RT + β-Lap/Fe NPs, RT + β-Lap/Fe NPs + αPD-L1. The mice were injected intravenously with β-Lap/Fe NPs and at 2 h post injection, the primary tumors of the mice were irradiated with 2 Gy X-ray. Antibodies were given at the day after the injection of β-Lap NPs by intraperitoneal injection at a dose of 50 µg per mouse. Specifically, β-Lap/Fe NPs were injected on day 0, day 2, day 4, day 6 and day 8. And αPD-L1 antibody was given on day 1, day 3, day 5, day 7, and day 9. The length and width of both primary tumors and distant tumors were also measured every other day.

For analyzing the activation of systemic antitumor immunity, the mice receiving different treatments were sacrificed on day 12 and the tumor tissues of the mice were collected. Flow cytometry is used to detect immune cells, the flow cytometry staining procedure was conducted as mentioned above.

### Statistical Analysis

All the statistical analysis was performed using GraphPad Prism 7.0. The statistical data were presented as Mean + SD. The student's t-test was used to analyze the differences between the two groups. A statistical significance of P < 0.05 was selected, and * indicated P < 0.05, ** indicated P < 0.01, and *** indicated P < 0.001, respectively.

## Results and discussion

### The preparation and characterization of β-Lap/Fe NPs

**Figure 1:**
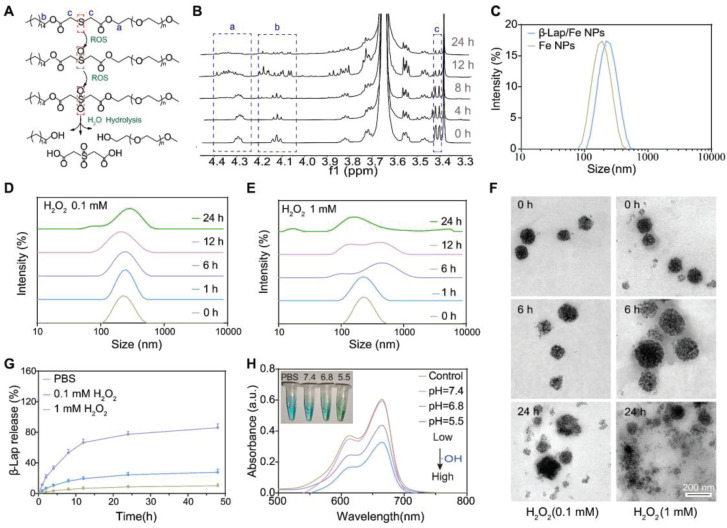
** The characterization of β-Lap/Fe NPs.** A) The simulation of oxidative degradation process of C16-S-mPEG2000. B) The characterization of oxidative degradation of C16-S-mPEG2000 with 1H NMR. C) The size distribution of β-Lap/Fe NPs and Fe NPs. The Changes in hydrodynamic size of β-Lap/Fe NPs treated with 0.1 mM (D) and 1 mM (E) H2O2 for different time. F) TEM images of β-Lap/Fe NPs before and after treated with 0.1 mM (left) and 1 mM (right) H2O2 for different time. G) The release of β-Lap from β-Lap/Fe NP treated with or without H2O2. H) The production of ·OH by β-Lap/Fe NP in 1 mM H2O2 with different pH value.

The ROS-responsive amphiphilic polymer was synthesized through a modified procedure from our previously reported method 42. As depicted in Figure S1A, the thiodiglycolic anhydride (THA) was separated in conjunction with 1-hexadecanol (C_16_-OH) and methoxypolyethylene glycol (mPEG_2000_) by esterification to form C_16_-S-mPEG_2000_. The chemical structure of intermediate product C_16_-S-COOH and end-product C_16_-S-mPEG_2000_ was characterized and confirmed by ^1^H NMR (Figure S1B and 1C). It has been reported that the thioether bonds in polymers could be oxidized to form sulfone and further cause hydrolyzation of the adjacent ester bond in the presence of high-level H_2_O_2_, thereby facilitating polymers degradation. Accordingly, the simulation of the oxidative degradation process of C_16_-S-mPEG_2000_ was depicted in Figure 1A. Considering that thioether bonds undergo significant oxidation at H_2_O_2_ concentrations above 1 mM, we selected this concentration to evaluate the ROS-responsive behavior of C_16_-S-mPEG_2000_. The oxidation of thioether bonds and the degradation of C_16_-S-mPEG_2000_ in the presence of H_2_O_2_ (1 mM) were measured utilizing ^1^H NMR (Figure 1B). The signal intensity of -SCH_2_- (δ 3.41 ppm) and -CH_2_O (δ 4.12 ppm and δ 4.29 ppm) in C_16_-S-mPEG_2000_ decreased after incubation with H_2_O_2_ for 4 h and further weaken over time, which revealed the thioether bonds oxidation and the both ester bond hydrolysis, respectively. The results demonstrated that H_2_O_2_ could induce the degradation of C_16_-S-mPEG_2000_.

Then, the hydrophobic Fe_3_O_4_ nanoparticles were prepared according to our previously reported method 42. The size distribution and morphology of Fe_3_O_4_ NPs were measured by DLS and TEM, respectively. As depicted in Figure S2, the Fe_3_O_4_ NPs with average hydrodynamic size of 7.4 nm, were well-defined spherical nanoparticles. The β-lapachone-based nanoparticles (β-Lap/Fe NPs) were fabricated by co-encapsulation of β-lapachone (β-Lap) and Fe_3_O_4_ nanoparticles utilizing amphiphilic C_16_-S-mPEG_2000_ and mPEG-PCL (Mw = 5000 g·mol^-1^). The β-Lap/Fe NPs, fabricated from 5 mg β-Lap, 5 mg Fe_3_O_4,_ 50 mg C_16_-S-mPEG_2000_, and 10 mg mPEG-PCL were determined to contain 3.3% weight of β-Lap and 7.3% weight of Fe_3_O_4_. Fe NPs were prepared using the aforementioned materials without the addition of β-Lap, serving as a control. The energy dispersive spectrometer (EDS) spectra, TEM mapping images and X-ray photoelectron spectrometer (XPS) were applied to investigate the presence of Fe_3_O_4_, Figure S3. The EDS spectra and TEM mapping images revealed the presence of Fe and O at β-Lap/Fe NPs. Furthermore, in XPS spectra, the significant the binding energies of Fe^2+^ and Fe^3+^ were observed, demonstrating the existence of Fe^2+^ and Fe^3+^ in β-Lap/Fe NPs. These results affirmed the presence of Fe_3_O_4_ in β-Lap/Fe NPs.

The size and stability of β-Lap/Fe NPs and Fe NPs were measured by dynamic light scattering (DLS) (Figure 1c). The β-Lap/Fe NPs and Fe NPs with average hydrodynamic size of 124.6 nm and 115.3 nm, respectively, were colloidally stable after incubation with PBS at 4 °C for a week (Figure S4). Further, the stability of β-Lap/Fe NPs were respectively investigated in PBS, DMEM and Fetal Bovine Serum (FBS) at 37 °C. The DLS results revealed that β-Lap/Fe NPs were colloidally stable after incubation with DMEM and FBS at 37 °C for one week (Figure S5).

### The ROS-responsive behavior of β-Lap/Fe NPs

The hydrophilic shell of nanomicelles plays a vital role in maintaining their stability by providing steric hindrance and surface protection. The degradation or detachment of this hydrophilic layer compromises micellar stability, resulting in structural disruption and premature release of loaded drugs. To investigate the oxidation response of β-Lap/Fe NPs, we measured the changes in particle size and stability of β-Lap/Fe NPs in H_2_O_2_ (a prevailing ROS simulant) at concentrations of 0.1 mM and 1 mM, respectively. The DLS intensity distribution of β-Lap/Fe NPs gradually increased upon H_2_O_2_ stimulation (Figure 1D and E). Compared to low concentrations of H_2_O_2_, higher concentrations were more likely to decrease the stability of β-Lap/Fe NPs, inducing the aggregation of nanoparticles. The stability of β-Lap/Fe NPs significantly declined after incubation with 1 mM H_2_O_2_ for 6 h, as indicated by an increase in their hydrodynamic size from 124.6 nm to 253.1 nm. Meanwhile, upon 1 mM H_2_O_2_ stimulation, the average size and PDI of β-Lap/Fe NPs were positive to the incubation time. In contrast, the size of β-Lap/Fe NPs exhibited minor changes after co-incubation with 0.1 mM H_2_O_2_ for 24 h. To further investigate the stability of β-Lap/Fe NPs in H_2_O_2_, DLS number distribution of β-Lap/Fe NPs was measured, Figure S6. The stability of β-Lap/Fe NPs gradually decreased upon H_2_O_2_ stimulation, which revealed by the changes of size distribution. Compared to low levels of H_2_O_2_, higher concentrations were more likely to decrease the stability of β-Lap/Fe NPs. Within the initial 12 h, β-Lap/Fe NPs treated with 1 mM H_2_O_2_ contained a large number of large aggregates, likely due to H_2_O_2_ triggering a part of hydrophilic layer degradation or detachment, increasing the hydrophobicity of the nanoparticle surface, leading to large amount of β-Lap/Fe NPs aggregation. After treatment with 1 mM H_2_O_2_ for 24 h, most or all of the hydrophilic segments degraded or detached from β-Lap/Fe NPs, causing the collapse of β-Lap/Fe NPs, the Fe_3_O_4_ NPs release and the formation of small aggregates, as revealed by the appearance of small particle size peak.

The H_2_O_2_ responsive behavior of β-Lap/Fe NPs was further confirmed by the transmission electron microscopy (TEM) images (Figure 1F). The well-defined spherical nanoparticles of β-Lap/Fe NPs were observed from the TEM images in absence of H_2_O_2_. Upon stimulation with 1 mM H_2_O_2_, β-Lap/Fe NPs underwent swelling and collapse at 6 h, followed by Fe_3_O_4_ nanoparticle aggregation. Conversely, β-Lap/Fe NPs underwent gently swelling and collapse at 24 hours upon treated with 0.1 mM H_2_O_2_. The aggregation of β-Lap/Fe NPs induced by H_2_O_2_ was attributed to the oxidation of sulfur atoms in C_16_-S-PEG_2000_, leading to the hydrolysis of adjacent ester bonds. As the ester bonds hydrolyze, the separation of hydrophilic PEG segments from the surface of β-Lap/Fe NPs results in an increase in surface hydrophobicity, further promoting the aggregation of residual nanoparticles through hydrophobic interactions. The efficiency of drug release is negatively correlated with the colloidal stability of nanomicelles. Therefore, the release of β-Lap was observed in the presence of H_2_O_2_ (Figure 1G). After incubation with 100 μM H_2_O_2_ for 24 h, with only 21.6% of the total amount of β-Lap was released from β-Lap/Fe NPs. In contrast, β-Lap/Fe NPs released β-Lap at a fast rate in the presence of 1 mM H_2_O_2_, with 73.1% of the total amount of β-Lap was released after incubation for 24 h. These results demonstrated that β-Lap/Fe NPs exhibited oxidation-responsive behavior, undergoing rapid disintegration in the presence of a high concentration of H₂O₂.

Subsequently, the Fenton reaction performance of β-Lap/Fe NPs was investigated at an H_2_O_2_ concentration of 1 mM. Iron mediated Fenton reaction was characterized by the generation of •OH from H_2_O_2_. In order to measure the •OH generation ability of β-Lap/Fe NPs, methylene blue (MB) was exploited as a substrate, where the blue MB would be degraded by •OH into colorless products 43,44. At acidic medium, the blue solution of MB contained β-Lap/Fe NPs and 1 mM H_2_O_2_ gradually turned pale, and the absorbance decreased in the ultraviolet-visible (UV-vis) absorption spectrum at 665 nm (Figure 1H). This trend is directly proportional to the acidity of the solution, which may be due to the tendency of acidic solutions facilitate the release of Fe^2+^ from collapsed β-Lap/Fe NPs. These results indicated that in an environment where acidity and high-levels of H_2_O_2_ coexist, β-Lap/Fe NPs could effectively trigger the Fenton reaction and generate plenty of •OH.

### X-ray upregulated cellular NQO1 to potentiate β-Lap-mediated H_2_O_2_ generation

**Figure 2:**
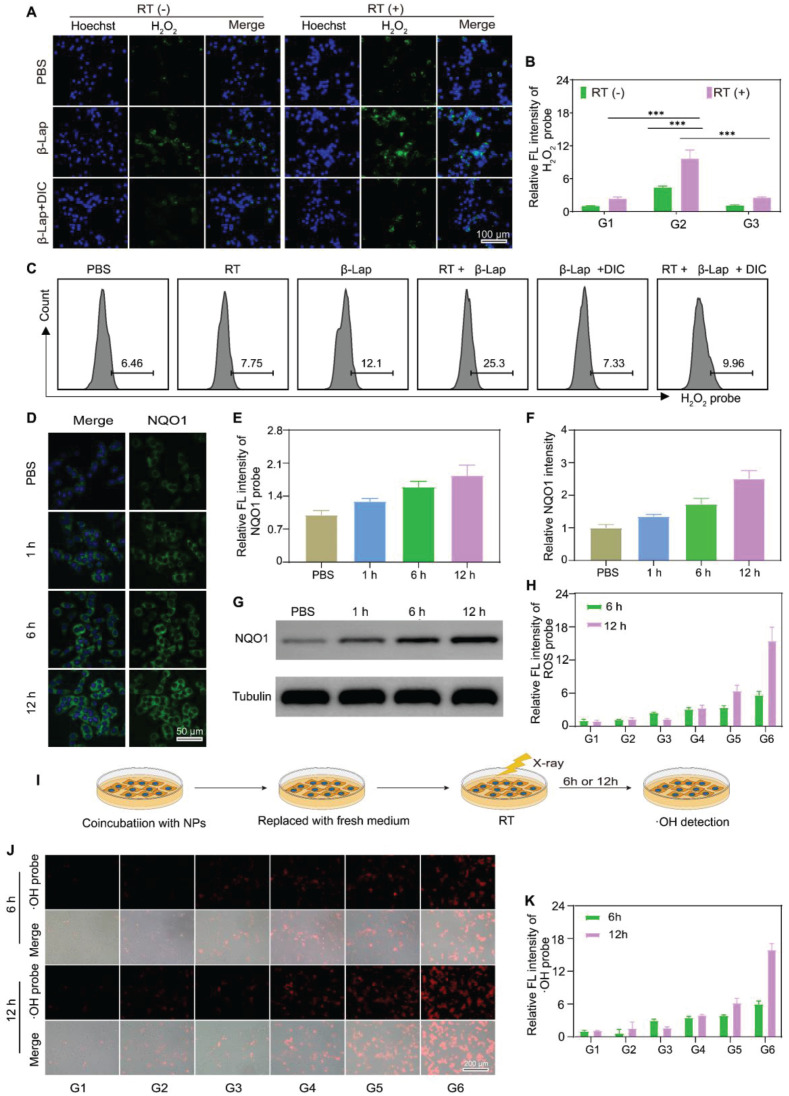
** X-ray irradiation promoted NQO1-dependent sustained ROS generation. IFM observed the production of H2O2 in 4T1 cells with different treatments.** (A) and corresponding relative fluorescence intensity (RFI) (B). G1: PBS, G2: β-Lap, G3: β-Lap + DIC (50 μM). C) FCM analysis of the production of H2O2 in 4T1 cells with different treatments. CLSM images of the expression of NQO1 in 4T1 cells after X-ray irradiation for different time (D) and corresponding RFI (E). Western blot analysis of the expression of NQO1 in 4T1 cells after X-ray irradiation for different time (G) and corresponding protein intensity (F). H) The RFL of production of ROS in 4T1 cells with different treatments. I) Schematic illustration of the persistent generation of •OH in 4T1 cells after different treatments. J) IFM observed the production of •OH in 4T1 cells sequentially incubated with NPs for 2 h and treated with or withouth X-ray irradiation and (K) corresponding RFI. G1: PBS, G2: β-Lap/Fe NPs, G3: RT, G4: RT + Fe NPs, G5: RT + β-Lap NPs, G6: RT + β-Lap/Fe NPs. Values are presented as the mean ± SD (n = 3). *P <0.05, **P < 0.01, ***P < 0.001.

β-Lap could be activated by NQO1, a cytosolic two-electron oxidoreductase that catalyzes quinones to the corresponding hydroquinones, resulting in forming H_2_O_2_ via NAD(P)H oxidation and leading to oxidative damage to tumor cells 45,46. However, β-Lap has shown unsatisfactory anti-tumor effects, largely attributed to the heterogeneous expression of NQO1 in tumor tissues. Recently, it has been reported that X-ray radiation could elevate the expression of NQO1 in tumor cells, reinforcing β-Lap cytotoxicity. Herein, we revealed that β-Lap could significantly elevate the level of H_2_O_2_ in 4T1 cells after X-ray radiation for 2 h. The intracellular H_2_O_2_ levels were measured by a H_2_O_2_ probe (BES-H_2_O_2_-Ac) using inverted fluorescence microscope (IFM) and flow cytometry (FCM), respectively. In the presence of H_2_O_2_, the BES-H_2_O_2_-Ac could be oxidized to emit green fluorescence. IFM images revealed that 4T1 cells treated with RT + β-Lap NPs exhibited significantly enhanced green fluorescence intensity compared to those treated with either RT or β-Lap alone (Figure 2A). Quantitative analysis showed that the green fluorescence intensity of cells treated with RT + β-Lap was 2.2-fold and 4.8-fold higher than that of cells treated with either RT or β-Lap alone, respectively (Figure 2B). Nevertheless, dicoumarol (DIC), an inhibitor of NQO1, dramatically impaired the H_2_O_2_ generation of RT + β-Lap as evidenced by the green fluorescence intensity in RT + β-Lap +DIC treated 4T1 cells was comparable to that in RT or β-Lap treated cells. Consistently, FCM results also revealed that RT + β-Lap could significantly reinforce the intracellular generation of H_2_O_2_ compared to others treatments, while dicoumarol sluggish this effect (Figure 2C). To further investigate the mechanism of upregulation of intracellular H_2_O_2_ upon treatment with RT + β-Lap we observed the NQO1 level by CSLM after RT radiation. Obviously, RT irradiation upregulated the intracellular level of NQO1, as indicated by increased green fluorescence of the NQO1 probe (Figure 2D). Typically, the expression of NQO1 was persistently upregulated for 12 h in RT treated cells, reaching a 1.95-fold higher level than in non-irradiated cells (Figure 2E). The X-ray triggered sustained upregulation of NQO1 was further confirmed by western blots analysis (Figure 2F-G). These results indicated that X-ray could potentiate the activity of β-Lap in tumor cells through the upregulation of NQO1, thereby promoting the generation of H_2_O_2_.

### X-ray stimulated β-Lap/Fe NPs to persistently generate •OH for strong cytotoxicity

The cellular uptake of β-Lap/Fe NPs was determined by measuring the intracellular concentration of iron ion utilizing inductively coupled plasma-mass spectrometry (ICP-MS). As depicted in Figure S7, the cellular concentration of iron ion was positively correlated with the coincubation time of β-Lap/Fe NPs and 4T1 cells. Therefore, we chose 2 h as the optimal uptake time for subsequent cell experiments. To investigate the anticancer performance of RT + β-Lap/Fe NPs, we first measured the intracellular ROS, which are associated with the anticancer efficiency of both CDT and RT (Figure S8-9 and Figure 2H). To detect ROS generation in 4T1 cells, ROS probe DCFH, which can be oxidized by ROS to emit green fluorescence, was utilized to stain pretreated 4T1 cells and observe with IFM. Although X-rays could enhance intracellular oxidative stress, the ROS levels in RT treated cells exhibited slight upregulation at the 6 h, and then returned to the initial state after irradiated for 12 h, exhibiting a weak oxidative stress state. Theoretically, RT induced upregulation of NQO1 could reinforced the oxidative stress of β-Lap, however, RT + β-Lap NPs gently enhanced the generation of ROS for 12 h compared to RT alone. This finding might be attributed to the low lethality and activity of H_2_O_2_, which could be quickly decomposed and neutralized by intracellular reducing system. Obviously, the DCF green fluorescence in 4T1 cells treated with RT + β-Lap/Fe NPs was the highest in comparison with other groups and could be sustained for 12 h, revealing the persistent generation of high levels of ROS, including but not limited to H_2_O_2_ and •OH (Figure 2H). To confirmed the intracellular generation of •OH, the IFM was applied to observed red fluorescence of oxidized BBoxiProbe®O28, which is a specific probe for •OH (Figure 2I). Undoubtedly, 4T1 cells treated with RT + β-Lap/Fe NPs generated highest level of •OH, which could persist for a duration of 12 h (Figure 2J-K). However, the generation of •OH in 4T1 cells separately treated with RT, β-Lap/Fe NPs, and RT + Fe NPs was negligible, which was consistent with the generation of H_2_O_2_. Additionally, it was noteworthy that β-Lap/Fe NPs treated 4T1 cells generated negligible ROS, including •OH, indicating its high stability and hypotoxicity. These results suggested that X-ray-induced upregulation of intracellular ROS could promote the degradation of β-Lap/Fe NPs and the release of Fe^2+^ and β-Lap, triggering a chain reaction that leads to the persistent production of highly reactive and lethal •OH through the Fenton reaction.

**Figure 3:**
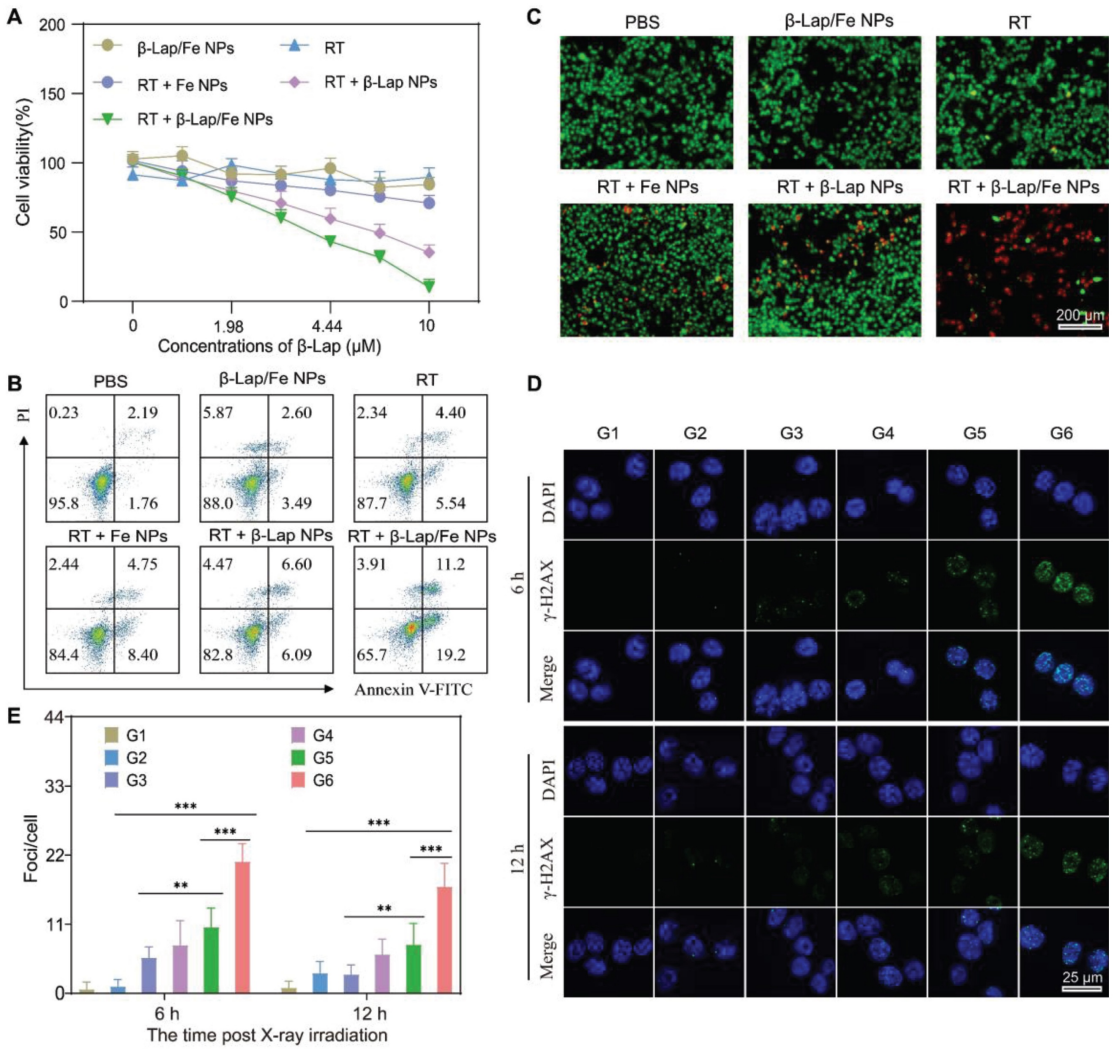
** The cytotoxicity of the combination of RT and β-Lap/Fe NPs.** A) MTT method analysis the viability of 4T1 cells incubated with β-Lap/Fe NPs for 2 h and treated with or without X-ray irradiation (2 Gy). B) FCM analysis of the apoptosis of 4T1 cells incubated with β-Lap/Fe NPs for 2 h and treated with or without X-ray irradiation (2 Gy). C) Live-dead staining of 4T1 cells incubated with β-Lap/Fe NPs for 2 h and treated with or without X-ray irradiation (2 Gy). The CLSM images (D) and corresponding mean fluorescence intensity of γ-H2AX staining in 4T1 cells incubated with β-Lap/Fe NPs for 2 h and treated with or without X-ray irradiation (2 Gy) G1: PBS, G2: β-Lap/Fe NPs, G3: RT, G4: RT + Fe NPs, G5: RT + β-Lap NPs, G6: RT + β-Lap/Fe NPs. Values are presented as the mean ± SD (n = 3). *P < 0.05, **P < 0.01, ***P < 0.001.

To investigate the performance of RT + β-Lap/Fe NPs in cancer cell inhibition, the MTT method was first applied to measure the cell viability. The RT + β-lap/Fe NP dramatically inhibited 4T1 cells viability may due to the strong oxidative damage caused by the persistent generation of •OH (Figure 3A). The cell viability inhibition rate of RT + β-Lap/Fe NPs was 8.7-fold higher than RT, 3.4-fold higher than RT + β-Lap NPs and 6.9-fold higher than RT + Fe NPs. Additionally, β-Lap/Fe NPs showed indiscernible toxicity may due to their high stability under the ROS level in 4T1 cells. Next, we utilized annexin V-FITC/PI double staining to evaluate cell apoptosis with flow cytometry assay (FCM) of each pretreated group (Figure 3B). Undoubtably, the percentage of apoptotic 4T1 cells treated with RT + β-Lap/Fe NPs was 30.4%, which was 3.1-fold, 5.0-fold, 2.4-fold, and 2.3-fold higher than that treated with RT, β-Lap/Fe NPs, RT + β-Lap NPs and RT + Fe NPs, respectively. Furthermore, the live-dead cell staining experiment was conducted to evaluate the cell inhibition effect of RT + β-Lap/Fe NPs (Figure 3C). Consistently, RT + β-Lap/Fe NPs induced highest levels of 4T1 cells dead than other groups, which was identified by the strong red fluorescence. At last, the results of γ-H2AX foci fluorescent staining, revealed that RT + β-Lap/Fe NPs could significantly reinforce DNA double-strand breaks in 4T1 cells after irradiation for 6 h (Figure 3D-E), which was 3.4-fold, 10.7-fold, 2.2-fold, and 2.4-fold higher than that treated with RT, β-Lap/Fe NPs, RT + β-Lap NPs and RT + Fe NPs, respectively. In contrast to the rapid repair of DNA damage in other groups, the strong DNA damage effect in RT + β-Lap/Fe NPs treated 4T1 cells could last for 12 h. All of the results revealed that X-ray stimulated β-Lap/Fe NPs to long-time generation of •OH, exhibiting strong therapeutic effects *in vitro*.

### X-ray initiated β-Lap/Fe NPs for potent tumor inhibition

Encouraged by the excellent therapeutic effects of RT + β-Lap/Fe NPs *in vitro*, we investigated its performance in animal models. Given that Fe_3_O_4_ NPs exhibit superparamagnetic effect, the tumor accumulation of β-Lap/Fe NPs was evaluated by magnetic resonance imaging (MRI). Additionally, the non-ROS-responsive N-β-Lap/Fe NPs were fabricated by the co-encapsulation of Fe_3_O_4_ NPs and β-Lap with amphiphilic C_16_-mPEG_2000_.

**Figure 4:**
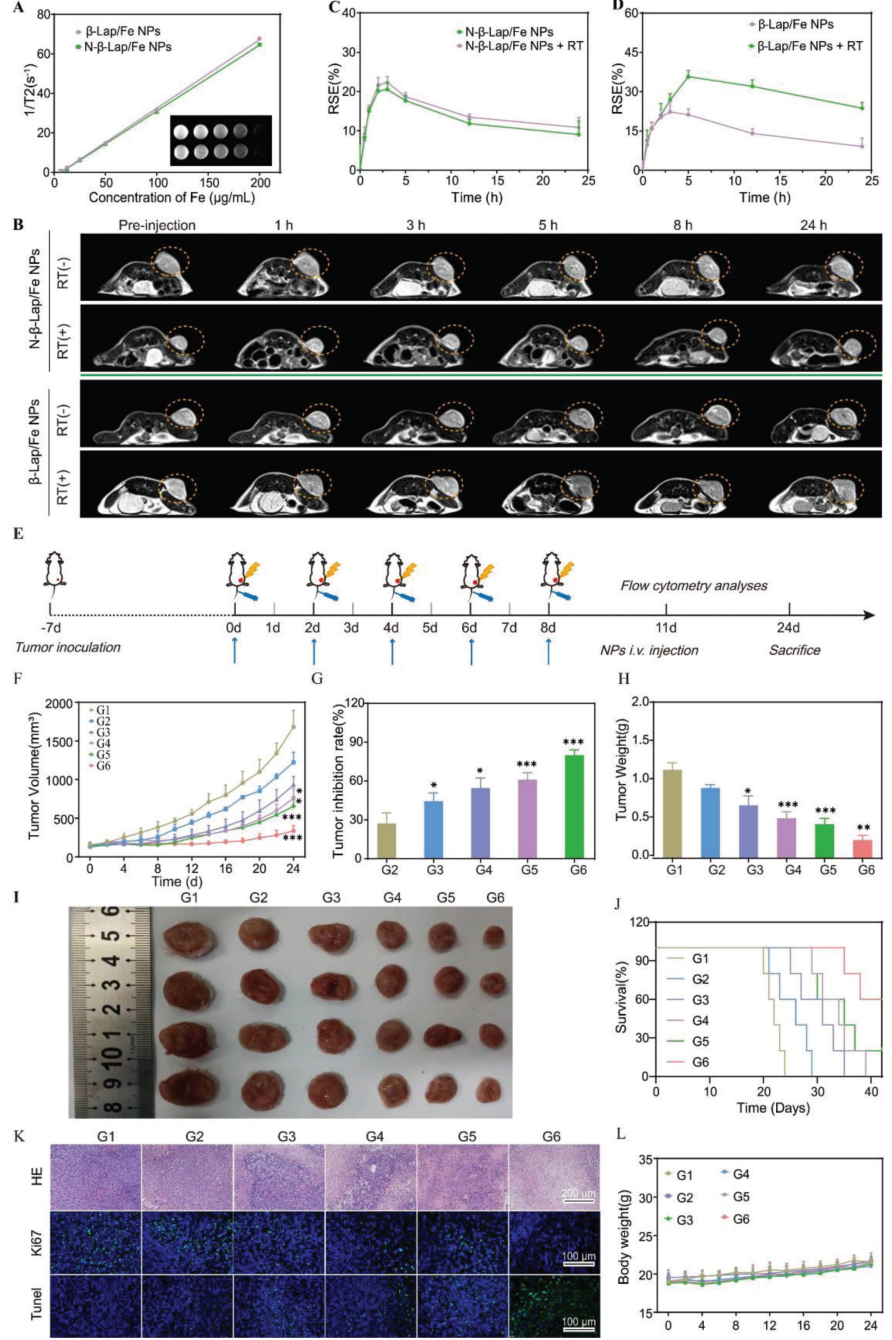
***In vivo* imaging and antitumor performance.** A) T2-weighted magnetic resonance images and transverse relaxation rates (1/T2) of β-Lap/Fe NPs and N-β-Lap/Fe NPs. B) *In vivo* axial T2-weighted magnetic resonance images and corresponding relative signal enhancement (%) (C and D) of 4T1 tumor-bearing mice at predetermined time. At 2 h post i.v. injection of β-Lap/Fe NPs and N-β-Lap/Fe NPs, the tumors were irradiated with X-ray at a dose of 2 Gy. Values are presented as the mean ± SD (n = 3). E) Schematic diagram of *in vivo* therapeutic schedule. Tumor growth curves (F) and tumor inhibition rate (TIR, %) (G) of 4T1 tumor-bearing mice under different treatments. H) The weight of tumors harvested on 24th day after different treatments. I) Representative photographs of 4T1 tumors harvested on 24th day after different treatments. J) Survival curves of tumor-bearing mice under different treatments. K) Immunofluorescence and H&E staining of tumor sections on 24th day after different treatments. L) The curves of body weight changes over time in 4T1 tumor-bearing mice under different treatments. G1: PBS, G2: β-Lap/Fe NPs, G3: RT, G4: RT + Fe NPs, G5: RT + β-Lap NPs, G6: RT + β-Lap/Fe NPs. Values are presented as the mean ± SD (n = 5). *P <0.05, **P < 0.01, ***P < 0.001.

Contributing to the T2-weighted (T2 WI) signal of N-β-Lap/Fe NPs and β-Lap/Fe NPs was positively correlated with their concentration (Figure 4A), the tumor accumulation of N-β-Lap/Fe NPs and β-Lap/Fe NPs could be measured with the T2 WI signal. As expected, the tumor accumulation of β-Lap/Fe NPs and N-β-Lap/Fe NPs could be observed post intravenously injected into 4T1 tumor-bearing mice for 0.5 h (Figure 4B), reaching its peak at 2 h. Additionally, after injection of β-Lap/Fe NPs for 24 h, the T2 WI signal maintain at 36.8% compared to the peak signal, thus revealing the perfect performance of β-Lap/Fe NPs in tumor accumulation and retention (Figure 4C-D). Moreover, the T2 WI signal in tumors treated with β-Lap/Fe NPs was comparable to that in tumors treated with N-β-Lap/Fe NPs, thus revealing that the ROS levels in tumors was insufficient for rapidly disintegrating β-Lap/Fe NPs. Although the concentration of ROS, including H_2_O_2_, at tumor sites is approximately 0.1 mM, its local concentration could increase beyond 20-fold after X-ray irradiation. To investigate whether X-ray-triggered upregulation of intratumor ROS could promote β-Lap/Fe NPs collapse, the tumors were irradiated with RT at a dose of 2 Gy after *i.v.* injection of β-Lap/Fe NPs for 2 h. After* i.v.* injection for 2 h, the T2 WI signal exhibited similar increasing trends in tumors treated with β-Lap/Fe NPs and N-β-Lap/Fe NPs, respectively. Compared to N-β-Lap/Fe NPs treated tumors, the T2 WI signal in tumors treated with β-Lap/Fe NPs gradually increased to 35.7% at 5 h post-injection, namely 3 h post-irradiation and exhibited slow recovery rate. As we previously reported, intratumor high level of ROS could trigger the cleavage of ROS-responsive linker and lead to detachment of PEG segment in micelles, contributing to the aggregation of Fe_3_O_4_ NPs and enhanced T2 WI signal. Therefore, these findings revealed that X-ray could promote disintegration of β-Lap/Fe NPs in tumors.

Subsequently, β-Lap/Fe NPs mediated tumor radio-chemodynamic therapy (RCD) was evaluated on 4T1 tumor-bearing mice, a model that exhibits early metastasis and high tumorigenicity, resembling human triple-negative breast cancer (TNBC). Once the tumor size reached nearly 150 mm^3^, BALB/C mice implanted with 4T1 cells on the right side of the flank were randomly divided into six groups, including G1: PBS, G2: β-Lap/Fe NPs, G3: RT, G4: RT + Fe NPs, G5: RT + β-Lap NPs, G6: RT + β-Lap/Fe NPs. The detailed treatment plan was the same as shown in the protocol (Figure 4E). The tumor volume and mouse weight were recorded every two days until any mouse tumor size reached 1500 mm^3^, a volume within the scope of animal ethics guidelines. Similar to the *in vitro* results, the treatment of β-Lap/Fe NPs exhibited no appreciable inhibitory effect on tumor growth, revealing its low cytotoxicity and high biosafety (Figure 4F). Although, the treatment of RT and RT + Fe NPs initially showed promise in slowing tumor growth at first 10 days, the tumors began to grow uncontrollably. Furthermore, we found that there was no significant difference in the tumor inhibition rate (TIR, %) (44.6% and 54.8%) and tumor weight (TW) (0.67 g and 0.51 g) between the two treatment groups (Figure 4G-H). It revealed that the intratumor H_2_O_2_ produced by X-ray was at a low level, making it difficult to activate efficient CDT. Conversely, RT + β-Lap NPs treatment moderately enhanced the TIR (61%) compared with alone RT treatment may be due to the production of high levels of H_2_O_2_ in the addition of β-Lap. However, due to the low lethality of H_2_O_2_, which can be easily decomposed or consumed by antioxidative mechanisms in tumors, RT + β-Lap NPs treatment showed lower TIR than RT + β-Lap/Fe NPs. Indeed, the treatment of RT + β-Lap/Fe NPs significantly inhibited tumor growth, exhibiting the highest TIR (80.3%) and lowest TW (0.23 g) than other groups. The highest tumor inhibition rate of RT + β-Lap/Fe NPs was also observed by the images of excised tumors (Figure 4I). Meanwhile, RT + β-Lap/Fe NPs treatment prolonged median survival time beyond 40 days, which was longer than 31, 34, and 36 days for the treatment of RT, RT + Fe NPs, and RT + β-Lap NPs (Figure 4J). The extraordinary efficiency of RT + β-Lap/Fe NPs in combating tumors could be attributed to the following two main steps. Firstly, after X-ray radiation, the enhanced tumor oxidative stress, including but not limited to H_2_O_2_, stimulated the destruction of β-Lap/Fe NPs and the release of β-Lap, further elevating the level of H_2_O_2_ through catalysis with X-ray-upregulated NQO1. Subsequently, the acidic microenvironment in tumor cells triggered the release of Fe^2+^ from Fe_3_O_4_ NPs, resulting in the continuous generation of •OH and long-time enhancement of oxidative stress by activating the Fenton reaction with high levels of H_2_O_2_.

Furthermore, the therapeutic effects were carefully investigated by conducting pathological examinations, including hematoxylin-eosin (H&E) staining, TdT-mediated dUTP nick-end labeling (TUNEL), and Ki67 staining (Figure 4K). The tumor slices collected from the mice treated with RT, RT + Fe NPs, and RT + β-Lap NPs respectively, exhibited slight tumor cell damage (H&E) and apoptosis (TUNEL), while those treated with RT + β-Lap/Fe NPs induced disastrous damage and apoptosis. Meanwhile, mice treated with RT + β-Lap/Fe NPs exhibited negligible tumor cell proliferation, characterized by the unobservable green fluorescence in Ki67 staining. Thus all, β-Lap/Fe NPs mediated radio-chemodynamic therapy could excellently inhibit tumor growth, meanwhile inducing negligible side effects (Figure 4L).

To investigate the biocompatibility of β-Lap/Fe NPs, the cytotoxicity on normal cells, hemolysis test, blood biochemical analysis and H&E histological analysis of major organs were conducted, Figure S10-S12. To evaluate the cytotoxicity of β-Lap/Fe NPs on normal cells, we firstly investigated the endogenous H_2_O_2_ level in 4T1 cells and normal cell lines L929 (mouse fibroblast cells). The endogenous levels of H_2_O_2_ in both non-cancerous and cancerous cells was quantitively measured using H_2_O_2_ assay kit according to the manufacturer's instructions, Figure S10.

Under comparable total cell quantities, the concentration of H_2_O_2_ in the 4T1 cells lysate is 21.4 μM, which is 112.8 times higher than that in L929 cells lysate, thus demonstrating the high H_2_O_2_ concentrations in cancer cells. Considering the minimal cytotoxicity of β-Lap/Fe NPs on 4T1 cells, it is expected that their cytotoxicity on L929 cells would also be negligible, as the low levels of ROS in normal cells make it challenging to trigger drug release from β-Lap/Fe NPs. The cytotoxicity of β-Lap/Fe NPs towards normal cells was measured by MTT methods. Normal cell lines L929 (mouse fibroblast cells) were separately incubated with different concentration of β-Lap/Fe NPs and free β-Lap. As depicted in Figure S11A, β-Lap/Fe NPs exhibited negligible toxicity to L929 cells, even at a β-Lap concentration of 10 μM, which was consistent with the negligible generation of ROS in L929 cells, Figure S11B. In contrast to β-Lap/Fe NPs, free β-Lap showed strong toxicity to L929 cells, with an IC_50_ of 6.34 μM. The blood biochemical analysis, including aspartate aminotransferase (AST), alanine aminotransferase (ALT), creatinine (CREA) and blood urea nitrogen (BUN) in β-Lap/Fe NPs treated mice remained within the normal range and exhibited negligible differences compared to PBS treated mice, Figure S11C. Lastly, there was negligible damage of cells observed in the heart, lung, kidney, and spleen, liver of the tumor-bearing mice at the last treatment for 3 days, Figure S11D. Consistently, the hemolysis test revealed that β-Lap/Fe NPs exhibited negligible hemolysis at different concentrations, Figure S12. These results demonstrated high biocompatibility of β-Lap/Fe NPs *in vitro* and *in vivo*.

### Mechanisms underlying the antitumor effects of β-Lap/Fe NPs mediated radio-chemodynamic therapy

**Figure 5:**
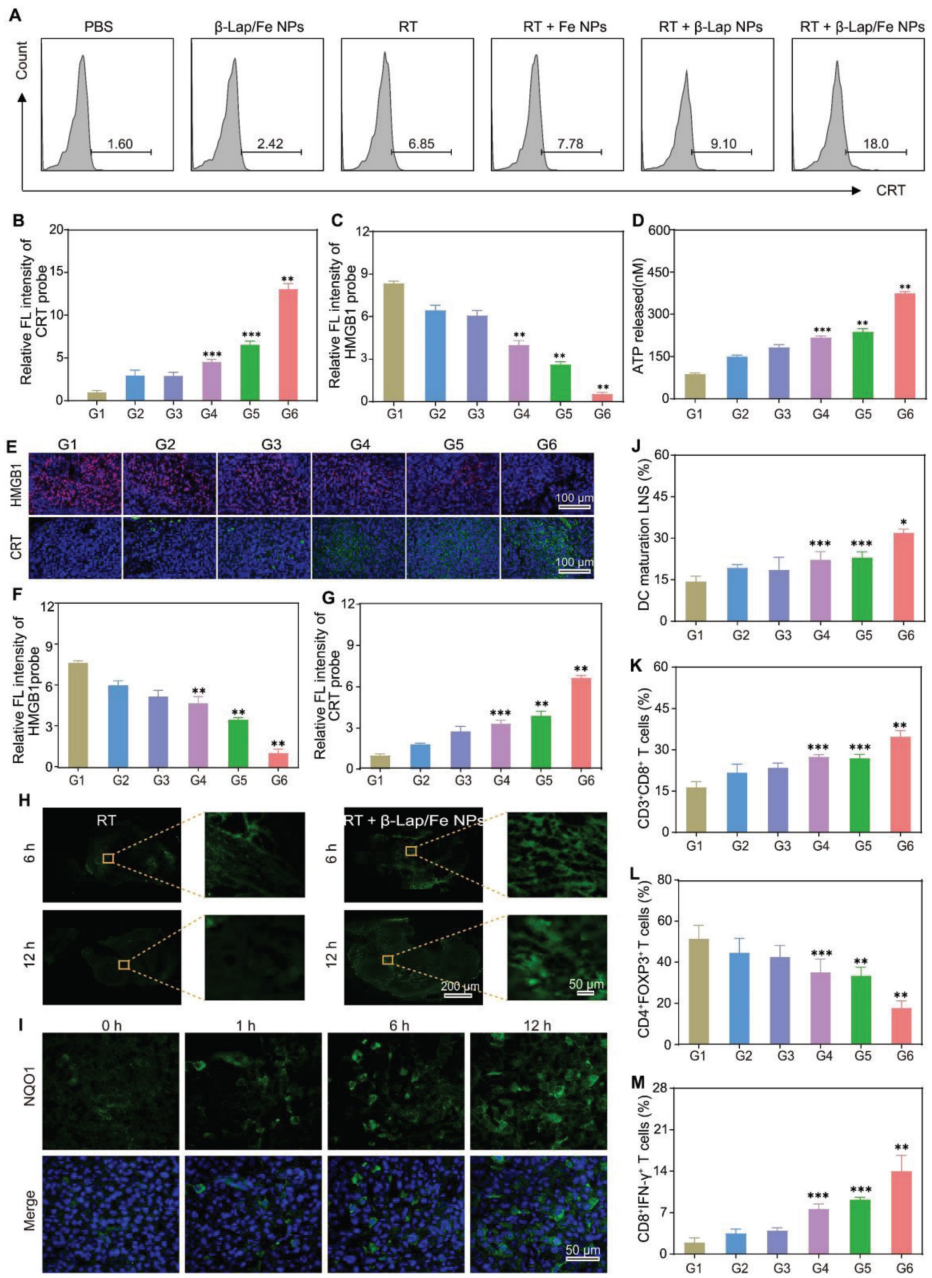
** The *in vitro* and *in vivo* ICD effects and immune activity.** A) The flow cytometry assay (FCM) analysis of the exposure of calreticulin (CRT) in 4T1 cells after different treatments. The relative intensity of CRT (B) and HMGB1 (C) observed by CLSM after different treatments. D) The release of ATP from 4T1 cells after different treatments. E) The HMGB1 and CRT immunofluorescence staining and corresponding intensity (F and G) of tumor sections on 24th day after different treatments. H) The ROS immunofluorescence staining of tumor sections after X-ray irradiation for 6 h and 12 h. I) The NQO1 immunofluorescence staining of tumor sections after X-ray irradiation for different times. J) The FCM analysis of DCs maturation in tumor-draining lymph nodes after last treatment for 3 days. Cells are gated by CD11c+ cells. The FCM analysis of (K) CD8+ T cells (gated by CD3+ T cells), (L) Tregs (gated by CD4+ T cells), and (M) CTLs (gated by CD8+ T cells) in tumor tissues 3 days after the last treatment. G1: PBS, G2: β-Lap/Fe NPs, G3: RT, G4: RT + Fe NPs, G5: RT + β-Lap NPs, G6: RT + β-Lap/Fe NPs. Values are presented as the mean ± SD (n = 3). *P <0.05, **P < 0.01, ***P < 0.001.

The immunogenic cell death (ICD) of tumor cells stands out as an excellent and safe process in producing endogenous immunologic adjuvant, which constructs the immunostimulatory microenvironment and antitumor immune network 47. ICD is characterized by the tumor-associated antigens (TAAs) and damage-associated molecular patterns (DAMPs), including but not limited to adenosine triphosphate (ATP), calreticulin (CRT), high mobility group box 1 (HMGB1). ATP, secreted from tumor cells, serves as a “find-me” signal to induce the accumulation of DCs. CRT, an endoplasmic reticulum resident protein, can transfer to the cell membrane surface when cells are subjected to exogenous/endogenous stimuli such as oxidative stress and promote recognition by DCs, acting as an “eat me” signal. HMGB1, a nuclear protein, can be released from the nucleus to the extracellular environment to promote DCs uptake and antigen presentation. The low oxidative stress caused by single X-ray irradiation made it difficult to damage the endoplasmic reticulum and nucleus, leading to the release or exposure of weak ATP, CRT, and HMGB1 *in vitro*, as depicted in Figure 5A-D and Figure S13 A-C.

As expected, RT + β-Lap/Fe NPs induced the strongest ICD effect compared to other groups, which was consistent with the generation of •OH. For example, the FCM results revealed that the exposure of CRT in RT + β-Lap/Fe NPs treatment was 2.6-fold and 2.0-fold higher than that of RT and RT + β-Lap NPs respectively (Figure 5A). Meanwhile, the release of ATP in RT + β-Lap/Fe NPs treatment was 1.7-fold higher than that of RT + Fe NPs treatment. Additionally, the treatment of RT + β-Lap/Fe NPs triggered strongest release of HMGB1 from 4T1 cell nucleus than other groups, as evidenced by the indiscernible red fluorescence in CLSM images and ELISA (Figure S13B-C). Therefore, the continuous generation of •OH stimulated by the combination of RT and β-Lap/Fe NPs was efficient for the induction of strong ICD *in vitro*.

The activation of ICD effects could positively induce dendritic cells (DCs) maturation, presenting the complex antigen peptide to the native T cell. To investigate the immunological effects of RT + β-Lap/Fe NPs towards bone-marrow-derived DCs, the costimulatory factor CD80^+^/CD86^+^ on DCs was measured by FCM. The DCs maturation was conducted using a transwell system, where the 4T1 cells upon different treatments and immature DCs were separately incubated in the upper and lower chamber. Consistent with the ICD effects, the levels of CD80^+^CD86^+^ DCs in RT + β-Lap/Fe NPs (26.5%) was highest than that in β-Lap/Fe NPs (9.94%), RT (11.9%), RT + Fe NPs (13%), RT + β-Lap NPs (16.2%), Figure S13D-E. Therefore, RT + β-Lap/Fe NPs could efficiently induce DCs maturation through the activation of strong ICD effects in* in vitro.*

Subsequently, the activation of ICD effects in the combination of RT and β-Lap/Fe NPs treatment *in vivo* was evaluated. After the last treatment for 3 days, the immunofluorescence assay was conducted to monitor the expression of CRT and release of HMGB1 *in vivo* (Figure 5E-G). The expression of CRT and the release of HMGB1 in RT + β-Lap NPs treated tumors was separately 1.4-fold and 1.5-fold higher than that in RT. Undoubtedly, the treatment of RT + β-Lap/Fe NPs triggered the highest expression of CRT, and release of HMGB1, which respectively reached 2.4-fold and 5.2-fold compared to RT, which could be attributed to the high level of ROS caused by this treatment (Figure 5H). Although the levels of ROS in RT treated tumors could upregulate at initial 6 h, it reduced to a negligible level at 12 h post X-ray irradiation. In contrast, the levels of ROS in RT + β-Lap/Fe NPs treated tumors maintained at a high level for 12 h, evidenced by the strong green fluorescence in tumor slices. Mechanically, RT irradiation could upregulate intratumor ROS and NQO1 (Figure 5I), and further initiated cascade reactions in conjunction with β-Lap/Fe NPs, persistently generating •OH over an extended period. Nevertheless, β-Lap/Fe NPs showed negligible influence on the expression of NQO1 in tumor tissues, Figure S14. This resulted in lethal oxidative stress and potent ICD effects *in vivo*.

The DAMPs released from dying tumor cells could recruit immature DCs to engulf the cancer cell corpses and TAAs, resulting in the maturation of DCs at the nearby lymph nodes and the presentation of processed antigens to native T cells. To investigate innate immune activation, the single-cell suspension of tumor-draining lymph nodes was conducted to analyze the DC maturation (CD80^+^CD86^+^) by FCM (Figure S15A). Due to the low expression of DAMPs, β-Lap/Fe NPs (19.9%), RT (17.7%), RT + Fe NPs (23%), RT + β-Lap NPs (24.5%) slightly triggered the maturation of DCs as compared with PBS (15.4%) (Figure 5J). Conversely, the treatment of RT + β-Lap/Fe NPs significantly induced the maturation of DCs (30.4%), which was consistent with the expression of DAMPs. Furthermore, the tumors treated with RT + β-Lap/Fe NPs reinforced the infiltration of CD3^+^CD8^+^ T cells (36.2%) that was 1.5-fold higher than RT (Figure 5K and Figure S15B). Although RT could enhance the population of CD4^+^ T cells, in which the infiltration of immunosuppressive CD4^+^ T cells (Tregs, CD4^+^Foxp3^+^) is dominant (39.5%), thereby exacerbating the tumor microenvironment immunosuppression (Figure 5L). Meanwhile, the immune microenvironment of tumors treated with β-Lap/Fe NPs exhibited negligible changes in compared with tumors treated with PBS. Inversely, RT + β-Lap/Fe NPs triggered a decrease of Tregs (CD4^+^Foxp3^+^, 21.6%) and an increase in CD8^+^ cytotoxic T lymphocytes (CD8^+^IFN-γ^+^, 16.8%) (Figure 5M). Consistently, in RT + β-Lap/Fe NPs, the level of inflammatory cytokines, including IFN-γ and TNF-α, were highest than other groups, Figure S16. Collectively, the combination of RT and β-Lap/Fe NPs triggered potent immunogenicity activation through the continuous generation of •OH. This process led to the improvement of the immunosuppressive environment and the infiltration of cytotoxic T cells in tumors.

### β-Lap/Fe NPs mediated RCDI against primary and distant tumors

Although radioimmunotherapy is currently being used in clinical practice for the treatment of certain cancers, the immunosuppressive tumor microenvironment and the low immunogenicity of X-rays greatly limit its effectiveness and widespread application. Given that the low level of oxidative stress induced by X-ray was correlated with the poor efficacy of radioimmunotherapy, we attempted to investigate whether β-Lap/Fe NPs could reinforce radioimmunotherapy. Firstly, we investigated the effect of β-Lap/Fe NPs mediated RCDI on tumor growth and metastasis with unilateral orthotopic 4T1 tumor models. To mimic human breast cancer, 4T1 cells expressing firefly luciferase (fLuc-4T1) were injected into abdominal mammary fat pads of female BALB/c mice to establish unilateral orthotopic 4T1 tumor models (Figure S17). Once the size of primary tumor reached 150 mm^3^, mice were randomly divided into five groups (n = 5), including: G1: PBS, G2: RT, G3: RT + αPD-L1, G4: RT + β-Lap/Fe NPs, G5: RT + β-Lap/Fe NPs + αPD-L1. The tumor growth and metastasis monitored using an *in vivo* fluorescence imaging system (IVFIS), Figure S17A. Not surprisingly, X-ray irradiation induced poor immunogenicity activation, RT + αPD-L1 just slightly inhibited primary characterized by the augmented bioluminescence signals at primary tumor sites. Conversely, RT + β-Lap/Fe NPs exhibited a stronger antitumor effect than RT + αPD-L1, revealed by the reduced bioluminescence signals at primary tumor sites. Furthermore, the treatment of RT + β-Lap/Fe NPs + αPD-L1 exhibited negligible bioluminescence signals at primary tumor sites, demonstrating the complete response of primary tumors. Furthermore, the bioluminescence imaging revealed that the lung metastasis started on the 16th day in the mice treated with PBS, RT and RT + αPD-L1. In contrast, the mice treated with RT + β-Lap/Fe NPs + αPD-L1showed negligible bioluminescence signals, indicating the tumor metastasis was significantly inhibited. The lung of mice in all groups were collected to directly evaluate the tumor metastasis, Figure S17B. The results of *ex vivo* bioluminescence imaging and gross nodules (Figure S17C) of lung showed significant tumor metastasis in the PBS, RT, and RT + αPD-L1 groups, while no detectable tumor metastases were observed in the RT + β-Lap/Fe NPs + αPD-L1 group. The HE staining of lung slices revealed that the lungs were occupied by tumors in the PBS, RT, and RT + αPD-L1 groups, while only a few minimal lung nodules were observed in RT + β-Lap/Fe NPs group. Significantly, negligible lung nodule was found in RT + β-Lap/Fe NPs + αPD-L1 group. These results demonstrated that β-Lap/Fe NPs mediated radio-chemodynamic-immunotherapy exhibited excellent performance in combating tumor growth and metastasis.

**Figure 6:**
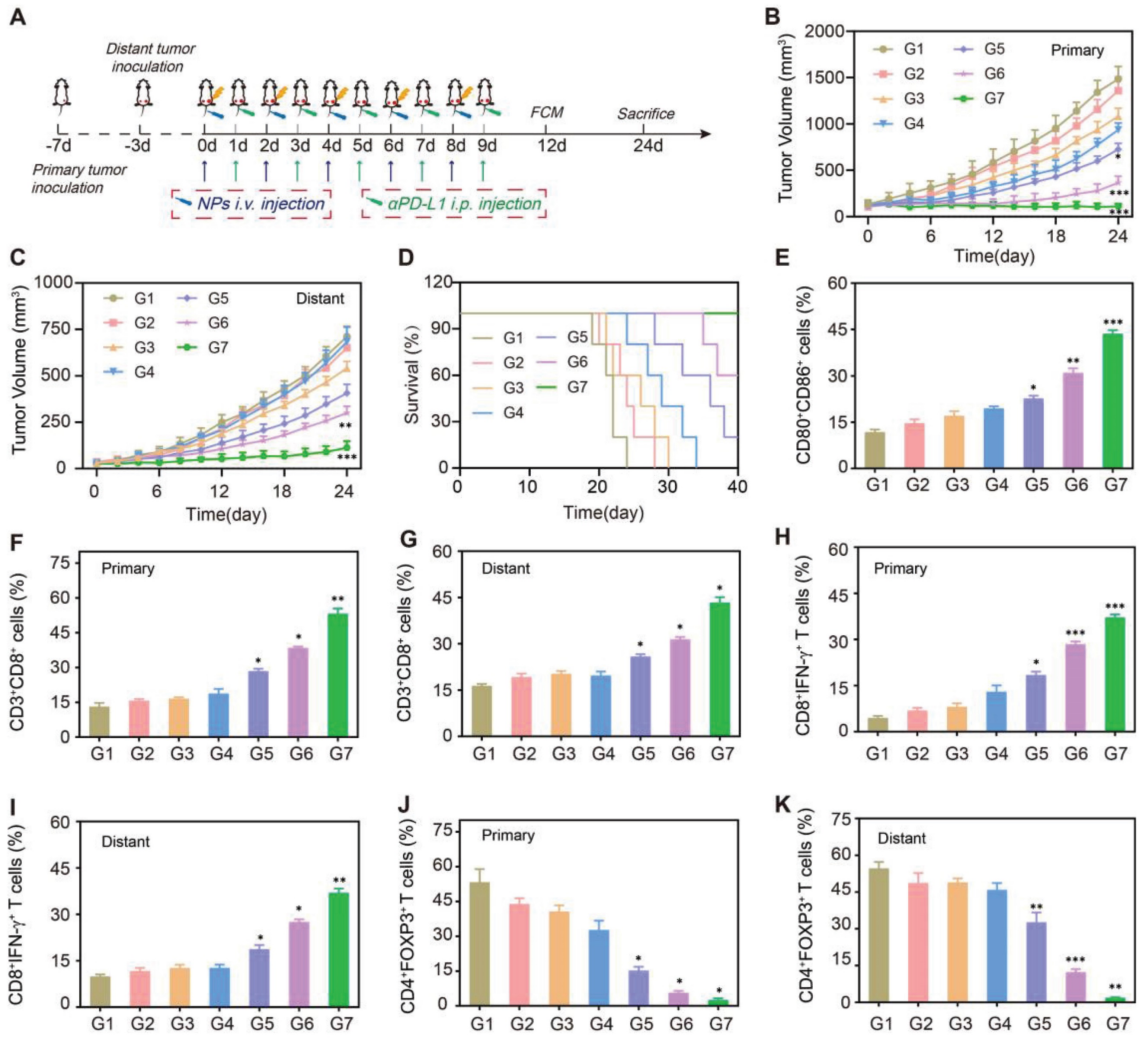
** The therapeutic performance in bilateral tumors.** A) Schematic diagram of *in vivo* therapeutic schedule. Primary tumor growth curves (B) and distant tumor growth curves (C) of 4T1 tumor-bearing mice under different treatments. D) Survival curves of tumor-bearing mice under different treatments. Values are presented as the mean ± SD (n = 5). *P < 0.05, **P < 0.01, ***P < 0.001. E) The FCM analysis of DCs maturation in tumor-draining lymph nodes adjacent to primary tumors after last treatment for 3 days. Cells are gated by CD11c+ cells. The FCM analysis of (F and G) CD8+ T cells (gated by CD3+ T cells), (H and I) CTLs (gated by CD8+ T cells), and Tregs (gated by CD4+ T cells) (J and K) in primary and distant tumor tissues after last treatment for 3 days. G1: PBS, G2: β-Lap NPs, G3: αPD-L1, G4: RT, G5: RT + αPD-L1, G6: RT + β-Lap/Fe NPs, G7: RT + β-Lap/Fe NPs + αPD-L1. Values are presented as the mean ± SD (n = 3). *P <0.05, **P < 0.01, ***P < 0.001.

Next, we established a bilateral 4T1 tumor-bearing mice model for evaluating the therapeutic effects on primary and distant tumor growth. After the primary tumors were inoculated at the right flank for 3 days, the second tumors were implanted at the left flank to mimic distant tumors (Figure 6A). Once the size of primary tumor reached 150 mm^3^, mice were randomly divided into seven groups (n = 5), including: G1: PBS, G2: β-Lap NPs, G3: αPD-L1, G4: RT, G5: RT + αPD-L1, G6: RT + β-Lap/Fe NPs, G7: RT + β-Lap/Fe NPs + αPD-L1. The representative photographs of mice in different groups and at different treatment time points were depicted in Figure S18.

In the absence of X-ray irradiation, the β-Lap NPs just induce negligible tumor inhibition on both primary and distant tumors, as the levels of ROS in tumor site is insufficient to triggered the β-Lap release. Although αPD-L1 could enhance the recognition of tumor cells by immune T cells *in vivo*, the low immunogenicity of 4T1 tumors makes it challenging to elicit an effective antitumor immune response. Therefore, the αPD-L1 showed slight inhibition on both primary and distant tumors. Not surprisingly, X-ray irradiation induced poor immunogenicity activation, RT + αPD-L1 just slightly inhibited primary and distant tumor growth (Figure 6B and C). Conversely, RT + β-Lap/Fe NPs exhibited a stronger abscopal antitumor effects than RT + αPD-L1. And, the effect of RT + αPD-L1 on primary and distant tumors was further reinforced upon addition of β-Lap/Fe NPs. Furthermore, the treatment of RT + β-Lap/Fe NPs + αPD-L1 led to complete response of primary tumors in 3 out of 5 mice and prolonged median survival time beyond 40 days, which was longer than RT, RT + αPD-L1, RT + β-Lap/Fe NPs (Figure 6D). To understand the therapeutic mechanism of β-Lap/Fe NPs mediated RCDI, the single-cell suspension of inguinal lymph nodes adjacent to primary tumors was collected for DCs analysis (Figure S19A). Although RT + αPD-L1 lightly reinforced DCs maturation (23.7%), the addition of β-Lap/Fe NPs exhibited significant effects on DCs maturation (42.2%) (Figure 6E).

The reinforced maturation of DCs in RT + β-Lap/Fe NPs + αPD-L1 treatment contributed to the intensified infiltration and activation of CD3^+^CD8 T^+^ cells in both primary (50.1%) and distant tumors (41.3%), which was 1.8 and 1.6 -folds higher than that in RT + αPD-L1 treatment, respectively (Figure 6F and G and Figure S19B). In the treatment of RT + β-Lap/Fe NPs, the level of CD3^+^CD8^+^ T in primary tumors upregulated to 2.0-fold higher than that in RT alone treatment. Additionally, in the treatment of RT + β-Lap/Fe NPs + αPD-L1, the level of CTLs (CD8^+^IFN-γ^+^ T cells) both upregulated in primary (17.1% to 36.0%) and distant tumor (18.6% to 35.6%) in contrast to RT + αPD-L1 treatment (Figure 6H and I and Figure S19C). Moreover, compared to the treatment of RT + αPD-L1, the improved immune microenvironments further revealed by the decrease of regulatory T cells (Tregs) (CD4^+^Foxp3^+^ T cells) in both primary (13.3% to 2.7%) and distant tumors (24.2% to 1.4%) in RT + β-Lap/Fe NPs + αPD-L1 treatment (Figure 6J-K and Figure S19D). Especially, RT + β-Lap/Fe NPs could reduce the level of Tregs to 8.6% in distant tumors. Consistently, in RT + β-Lap/Fe NPs, the level of inflammatory cytokines, including IFN-γ and TNF-α, were highest than other groups, Figure S20. Overall, these results demonstrated that X-ray could fixed point actuate β-Lap/Fe NPs to initiate potent systemic immune responses by inducing strong ICD effects, DCs activation, CTLs infiltration as well as the decreasing of Tregs, resulting in vigorous antitumor effects on primary and distant tumors in cancer RCDI.

## Conclusion

In conclusion, we exploited X-ray to locally and visually actuate β-lapachone-based nanoparticles (β-Lap/Fe NPs) to reinforce the immunogenicity of tumor cells by persistently generating **·**OH for MRI-guided cancer RCDI. β-Lap/Fe NPs were prepared from the co-loaded β-Lap and Fe_3_O_4_ nanoparticles with PEG_2000_-S-C_16_, in which, the thioether bond could be destroyed under high-level ROS induced by X-ray radiation, leading to the release of β-Lap and Fe^2+^. We found that upon X-ray irradiation, the intracellular upregulation of NQO1 positively catalyzed the released β-Lap to continuously generate plenty of H_2_O_2_, which was further transformed to highly toxic •OH through Fenton-reaction for 12 h, eliciting strong ICD effects *in vitro* and *in vivo*. Specifically, the sequentially enhanced T2 WI in tumors induced by the EPR effect and ROS guided accurate X-ray irradiation and predicted local generation of ·OH, respectively. Finally, X-ray locally actuated β-Lap/Fe NPs to elicit strong immune responses in combination with αPD-L1, significantly inhibiting primary tumor growth and exhibiting a strong abscopal antitumor effect. This work proposed a new method of immunogenic amplification, which involved using β-Lap-based nanoparticles to locally and visually generate •OH for a long-time under X-ray stimulation, is safe and versatile, and shows potential in combating tumors with poor immunogenicity, such as radioresistant tumors.

## Supplementary Material

Supplementary figures.
