# Supplementary material for: X-ray stimulates NQO1-dependent cascade reactions to induce strong immunogenicity for MRI-guided cancer radio-chemodynamic-immunotherapy
Source: Theranostics. 2025 Jun 9;15(14):6768–88. doi: 10.7150/thno.110573 (PMC12203676; doi:10.7150/thno.110573)
Supplement: Supplementary file 1 — Supplementary figures. [file thnov15p6768s1.pdf]

X-ray stimulates NQO1-dependent cascade reactions to induce strong immunogenicity for MRI-guided cancer radio-chemodynamic-immunotherapy

Li He[1,2,] Jiao-Jiao Ma[2,*], Yi-Qun Wu[1], Chen-Guang Wang[5], Tong Lan[5], Lu Su[5], Lin Zhu[2,✉], Shi-Wen Huang[3,4,✉], Kai Deng[2,✉], Yong-Chang Wei[1,✉]*

1. Department of Radiation and Medical Oncology, Zhongnan Hospital of Wuhan University, Wuhan 430071, China
2. Department of Radiology, Zhongnan Hospital of Wuhan University, Wuhan 430071, China
3. Key Laboratory of Biomedical Polymers of Ministry of Education, Department of Chemistry, Wuhan University, Wuhan 430072, China
4. Department of Orthopedic Trauma and Microsurgery, Zhongnan Hospital of Wuhan University, Wuhan 430071, China
5. Hubei Bio-Pharmaceutical Industrial Technological Institute Inc, Wuhan 430205, China

[*] These authors equally contributed to this work.

✉ Corresponding authors: zljessi730@126.com (L. Zhu), swhuang@whu.edu.cn (S.W. Huang), dengk@whu.edu.cn (K. Deng), weiyongchang@whu.edu.cn (Y.C. Wei).

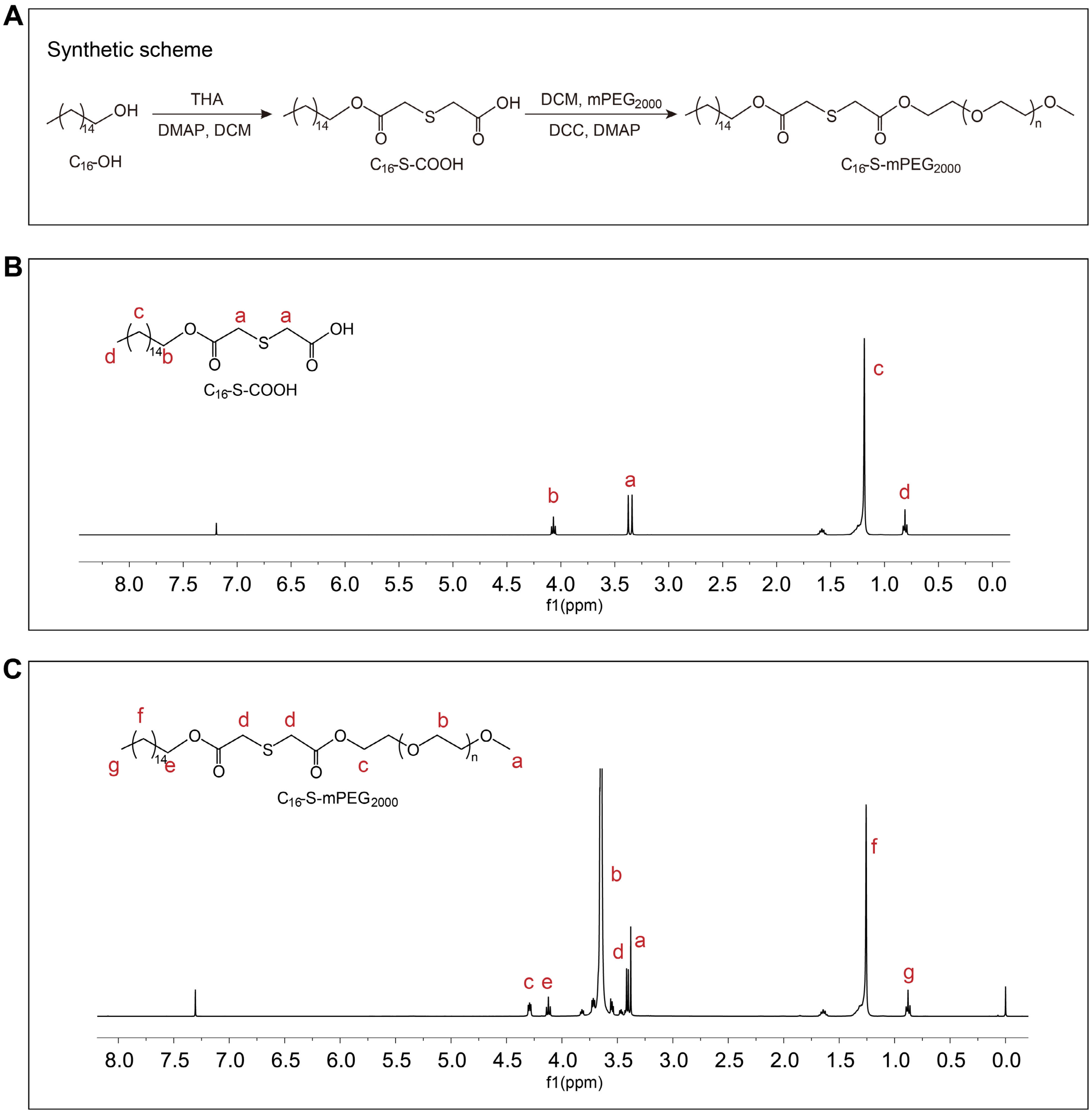

Figure S1. (A) Synthesis of C_{16}-S-COOH and $mPEG_{2000}$ -S- C_{16}. ^{1}H NMR spectra of C_{16}-S-COOH (B) and $mPEG_{2000}$-S-C_{16} (C).

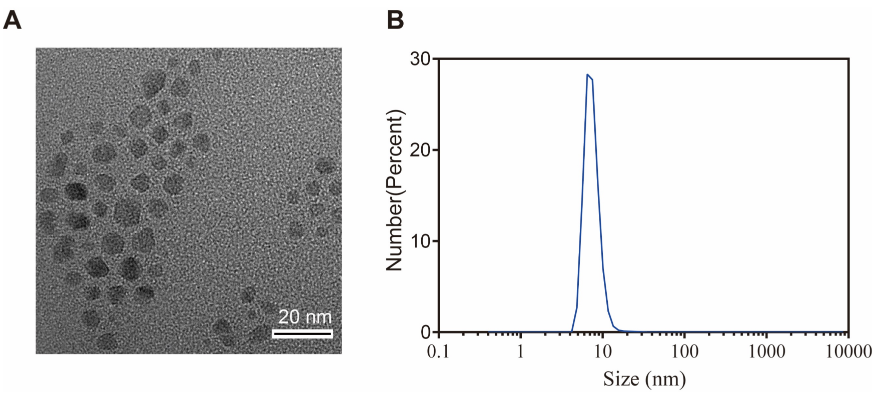

Figure S2. The TEM image and DLS result of Fe_3O_4 NPs.

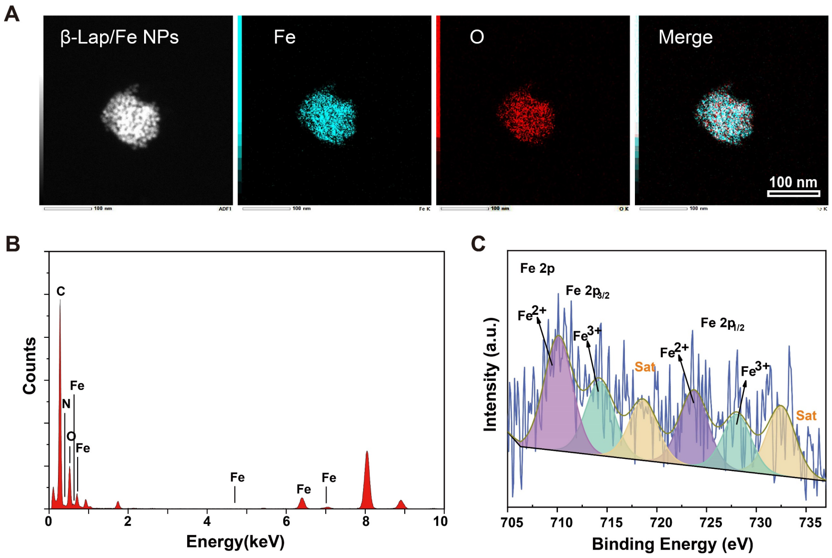

Figure S3. A) TEM mapping images, (B) EDS spectra, and (C) XPS spectra of the β-Lap/Fe NPs.

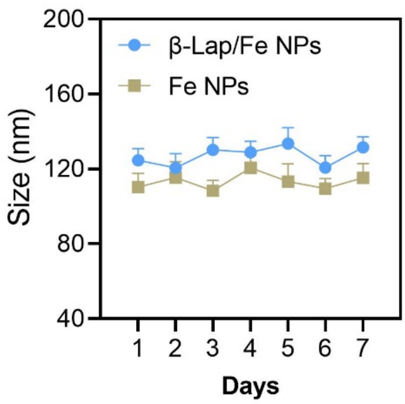

Figure S4. Size distribution of β-Lap/Fe NPs and Fe NPs during stored at 4 °C for one week.

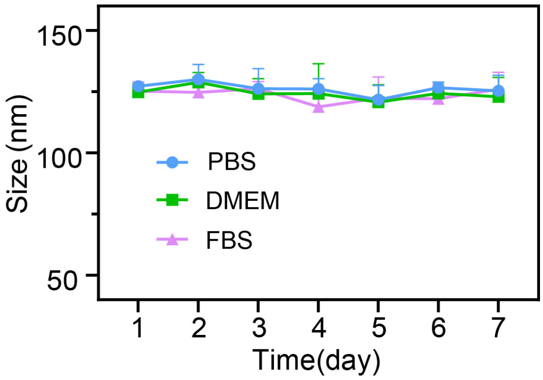

Figure S5. Size distribution of β-Lap/Fe NPs in PBS, DMEM and FBS during stored at 37°C for one week.

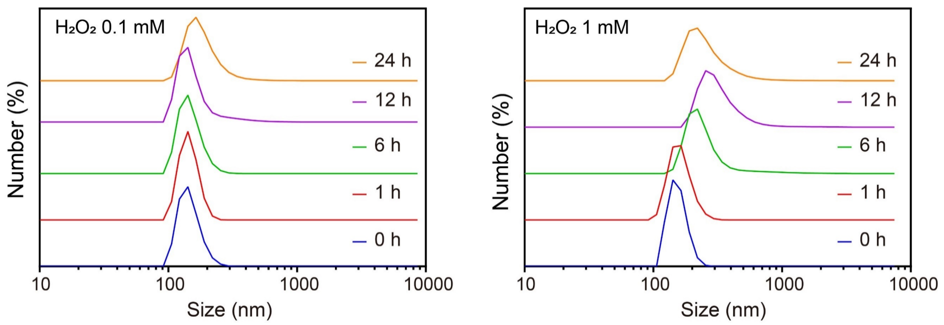

Figure S6. DLS number distribution of β-Lap/Fe NPs after treated with H_2O_2 for different time.

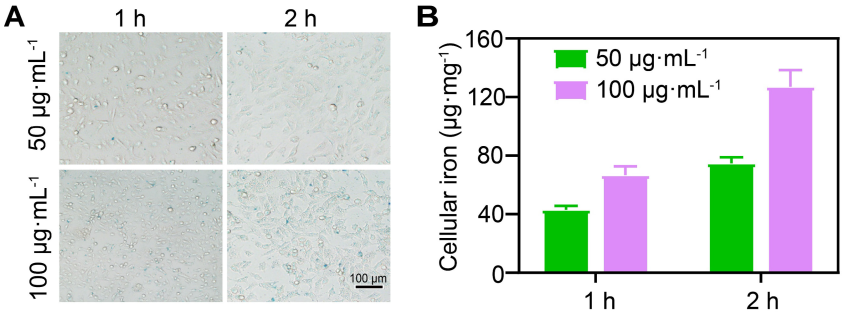

Figure S7. (A) Prussian blue staining of 4T1 cells incubated with β-Lap/Fe NPs for 1 h and 2 h at a concentration of 50 and 100 μg·mL^{-1} and corresponding iron concentration in 4T1 cells (B).

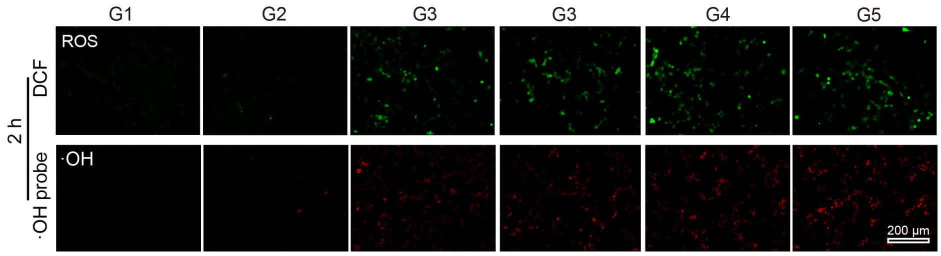

Figure S8. IFM observed the production of ROS and •OH in 4T1 cells post X-ray irradiation for 2 h. G1: PBS, G2: β-Lap/Fe NPs, G3: RT, G4: RT + Fe NPs, G5: RT + β-Lap NPs, G6: RT + β-Lap/Fe NPs.

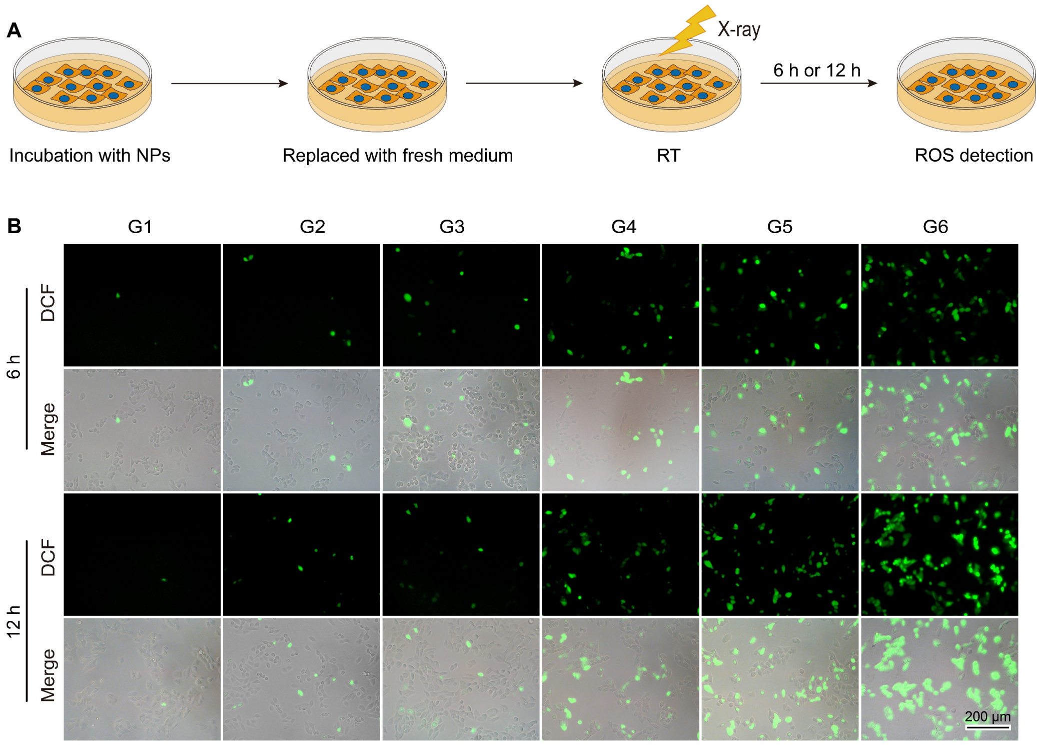

Figure S9. A) Schematic illustration of the persistent generation of ROS in 4T1 cells after different treatments. B) IFM observed the production of ROS in 4T1 cells sequentially incubated with NPs for 2 h and treated with or withouth X-ray irradiation. G1: PBS, G2: β-Lap/Fe NPs, G3: RT, G4: RT + Fe NPs, G5: RT + β-Lap NPs, G6: RT + β-Lap/Fe NPs.

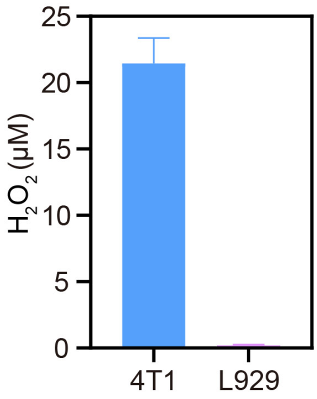

Figure S10. The concentration of H_2O_2 in the cell lysate of 4T1 cells and L929 cells.

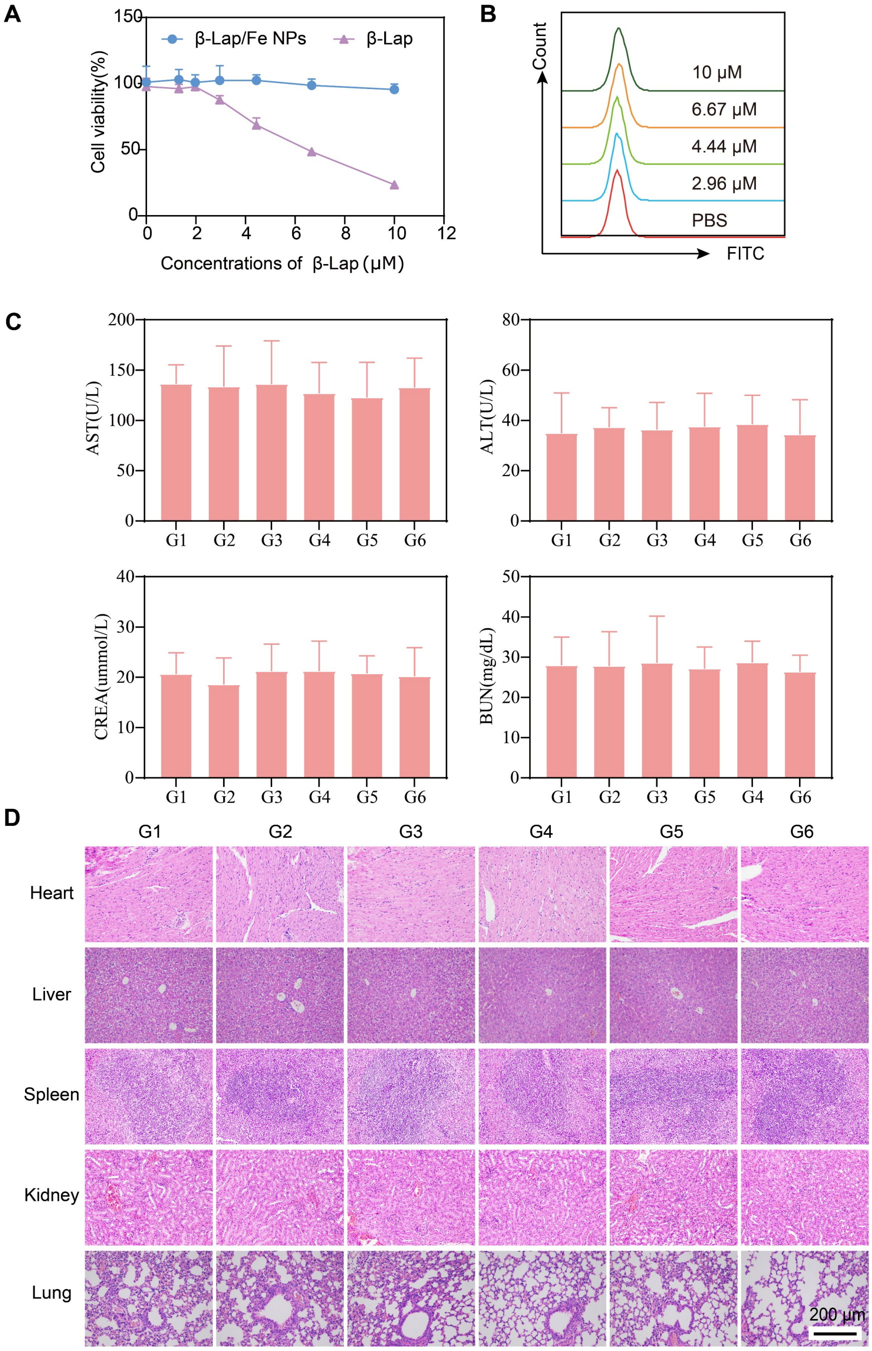

Figure S11. The biocompatibility of β-Lap/Fe NPs. (A) The cytotoxicity of β-Lap/Fe NPs towards Normal cell lines L929 measured using MTT method. (B) The generation of ROS in L929 cells after treatment with β-Lap/Fe NPs for 24 h determined by FCM. (C) The blood biochemical index test of mice after different treatments. G1: PBS, G2: β-Lap/Fe NPs, G3: RT, G4: RT + Fe NPs, G5: RT + β-Lap NPs, G6: RT + β-Lap/Fe NPs. (D) H&E staining of major organs, including heart, liver spleen, lung, and kidney of tumor-bearing mice collected at the last treatment for 3 days.

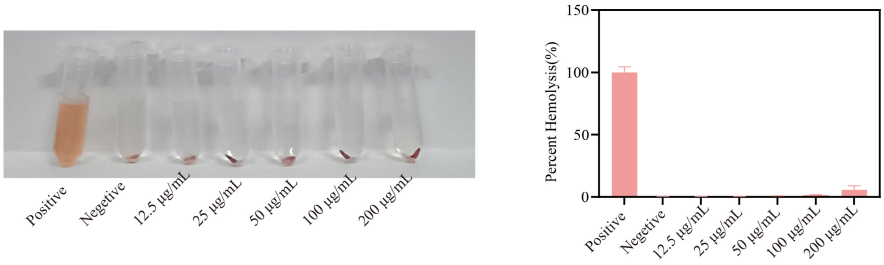

Figure S12. The hemolysis of β-Lap/Fe NPs at different concentration.

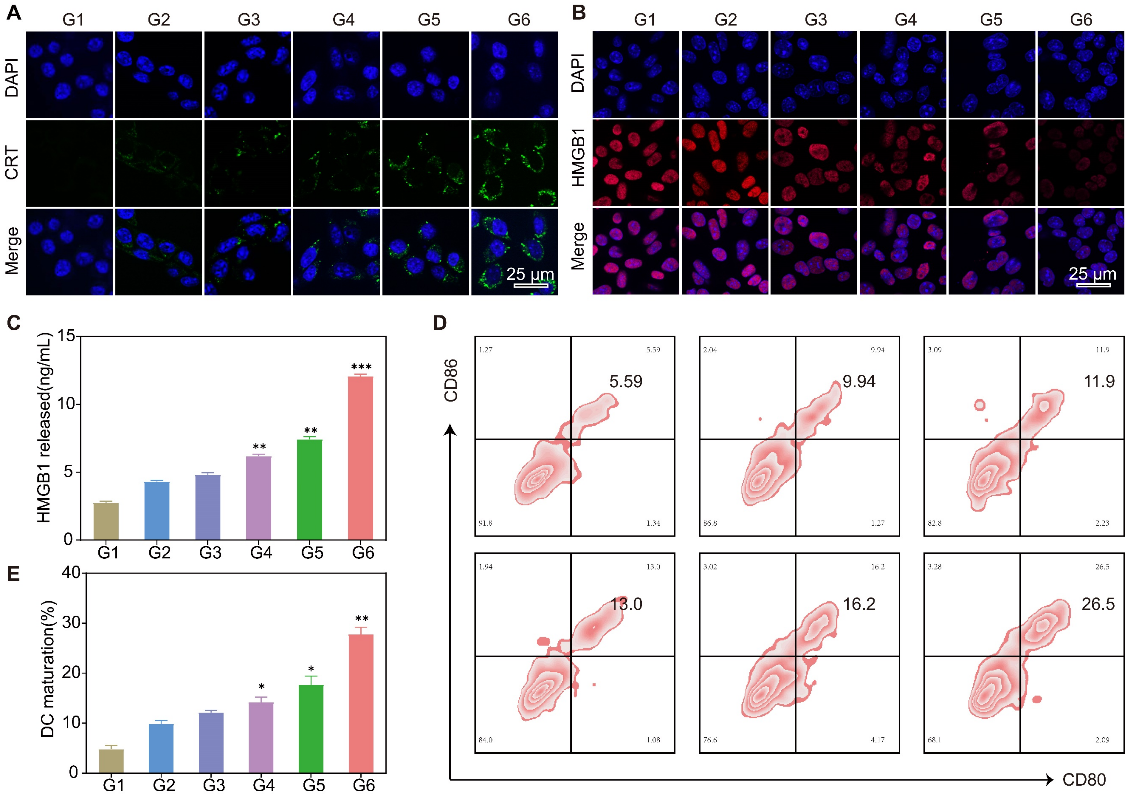

Figure S13. The *in vitro* ICD effects and DCs maturation. The CRT (A) and HMGB1 (B) observed by CLSM after different treatments. (C) The HMGB1 release was measured by ELISA after different treatments. (D and E) The DCs maturation analyzed using FCM after different treatments. G1: PBS, G2: β-Lap/Fe NPs, G3: RT, G4: RT + Fe NPs, G5: RT + β-Lap NPs, G6: RT + β-Lap/Fe NPs.

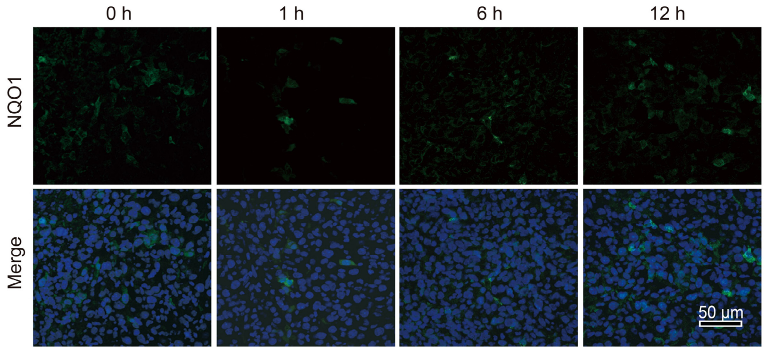

Figure S14. The NQO1 immunofluorescence staining of tumor sections after β-Lap/Fe NPs treatment for different time.

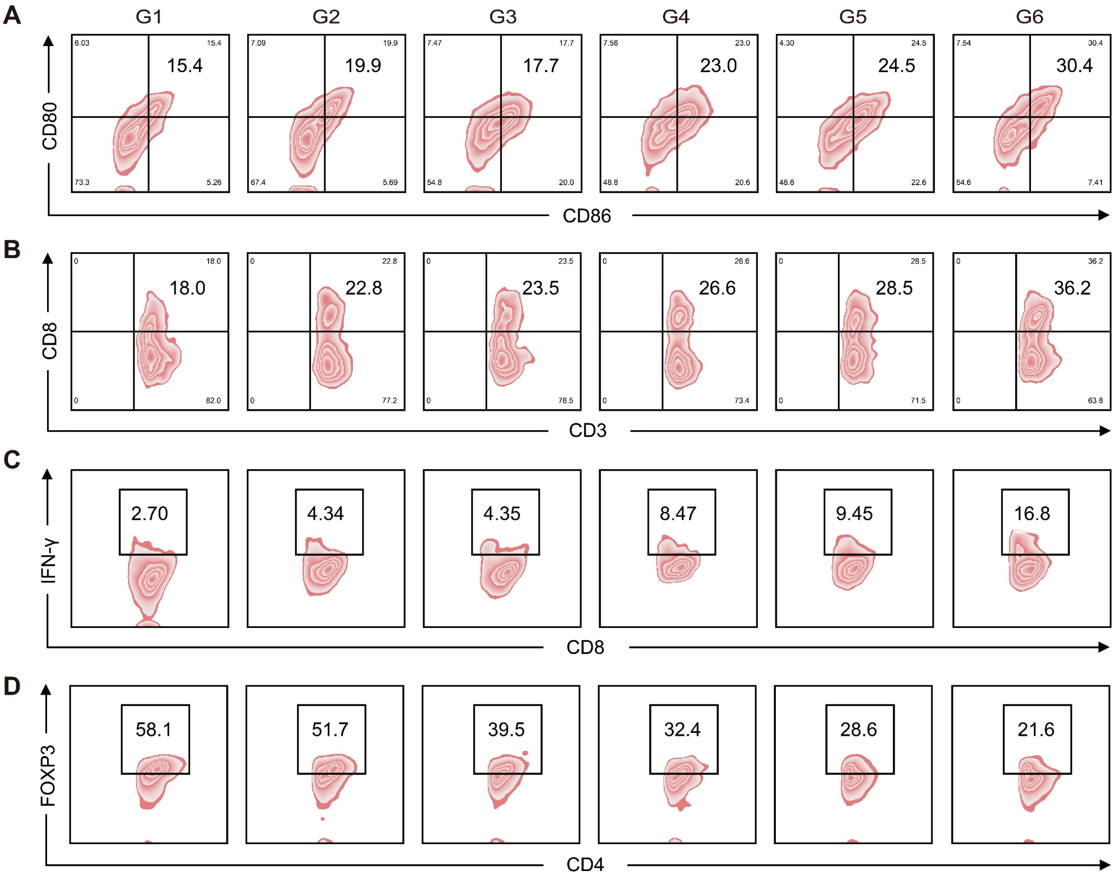

Figure S15. A) The FCM analysis of DCs maturation in tumor-draining lymph nodes after last treatment for 3 days. Cells are gated by $CD11c^+$ cells. The FCM analysis of (B) $CD8^+$ T cells (gated by $CD3^+$ T cells), (C) Tregs (gated by $CD4^+$ T cells), and (D) CTLs (gated by $CD8^+$ T cells) in tumor tissues after last treatment for 3 days. G1: PBS, G2: β-Lap/Fe NPs, G3: RT, G4: RT + Fe NPs, G5: RT + β-Lap NPs, G6: RT + β-Lap/Fe NPs.

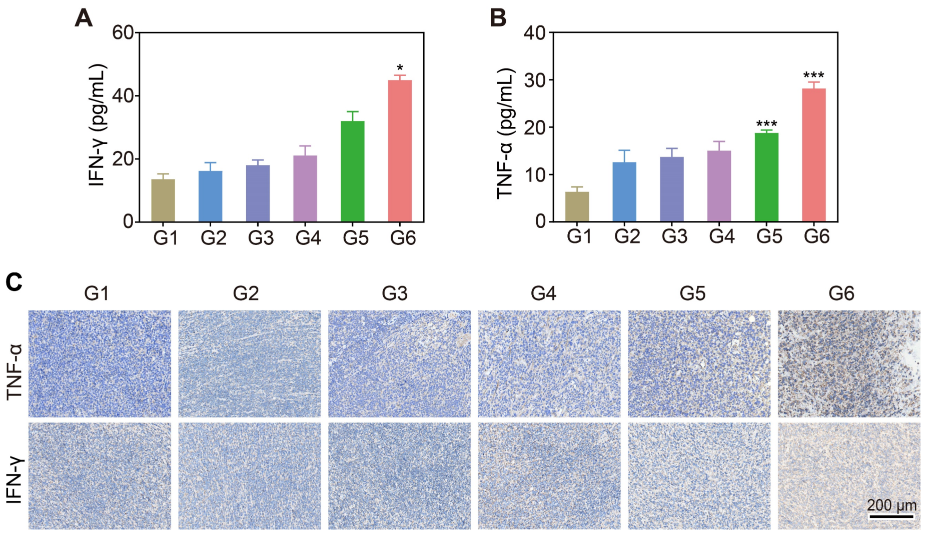

Figure S16. The levels of IFN-γ (A) and TNF-α (B) in unilateral 4T1 tumor-bearing mice serum isolated at last treatment for 3 days. (C) The immunohistochemical staining of tumor slices at last treatment for 3 days. G1: PBS, G2: β-Lap/Fe NPs, G3: RT, G4: RT + Fe NPs, G5: RT + β-Lap NPs, G6: RT + β-Lap/Fe NPs. Values are presented as the mean ± SD (n = 3). *P <0.05, **P < 0.01, ***P < 0.001.

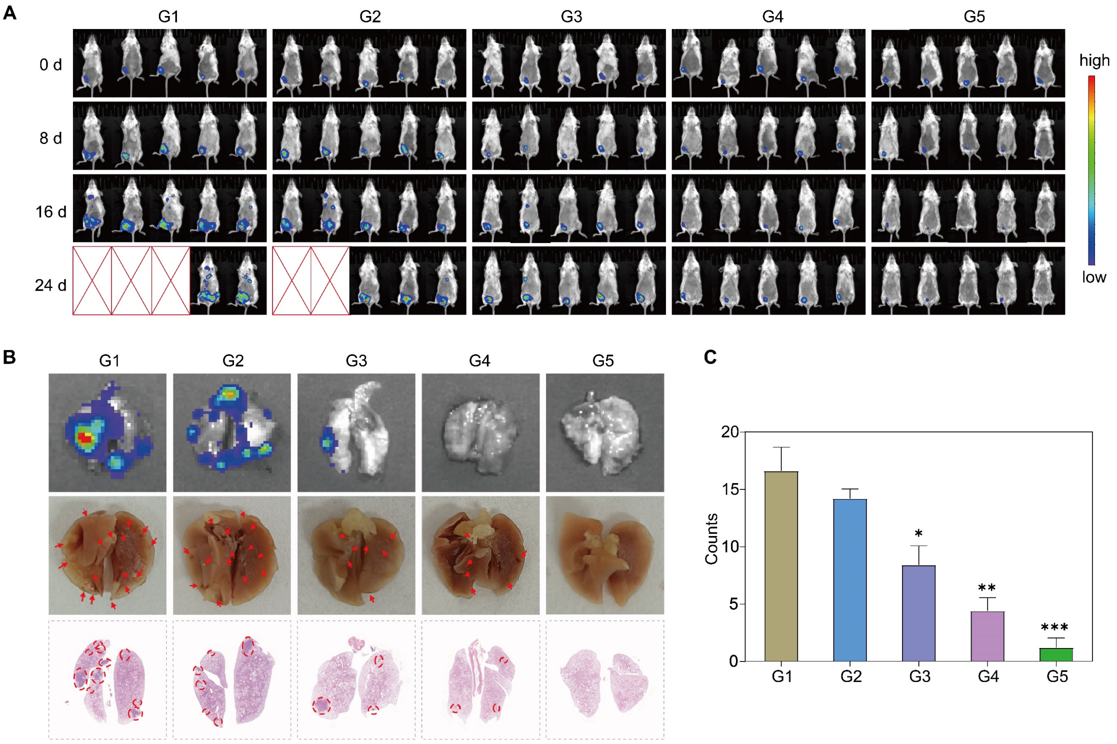

Figure S17. Antitumor effect on unilateral orthotopic 4T1 tumor models. (A) The in vivo bioluminescence images tracking the growth and metastasis of fLuc-4T1 tumors in the mice after different treatments. (B) representative ex vivo bioluminescence images, photographs, and HE staining of lung slices to show the of tumor nodules in the lungs. (C) the average numbers of lung nodules counted under anatomy microscope. G1: PBS, G2: RT, G3: RT + αPD-L1, G4: RT + β-Lap/Fe NPs, G5: RT + β-Lap/Fe NPs + αPD-L1. Values are presented as the mean ± SD (n = 3). *P <0.05, **P < 0.01, ***P < 0.001.

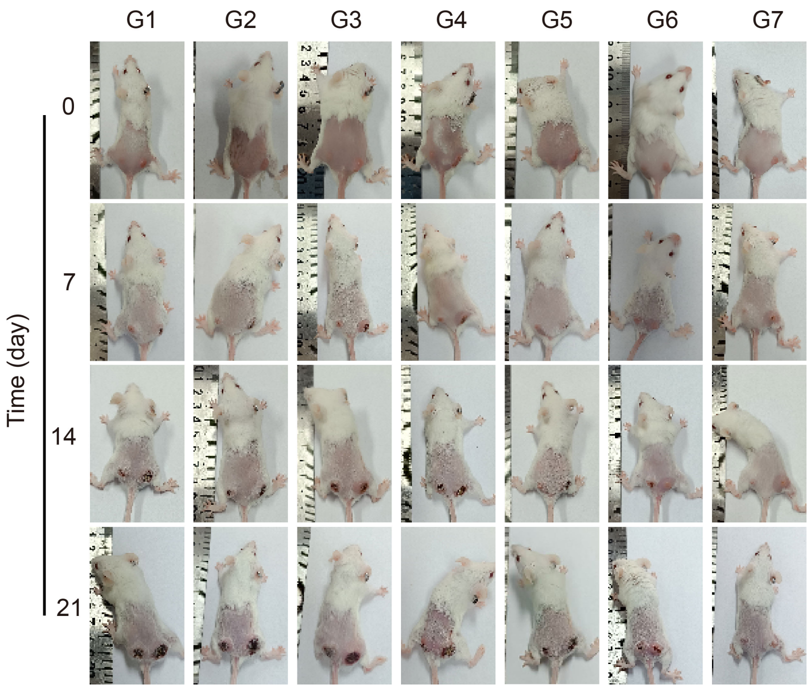

Figure S18. The representative photographs of mice in different groups and at different treatment time points. G1: PBS, G2: β-Lap NPs, G3: αPD-L1, G4: RT, G5: RT + αPD-L1, G6: RT + β-Lap/Fe NPs, G7: RT + β-Lap/Fe NPs + αPD-L1.

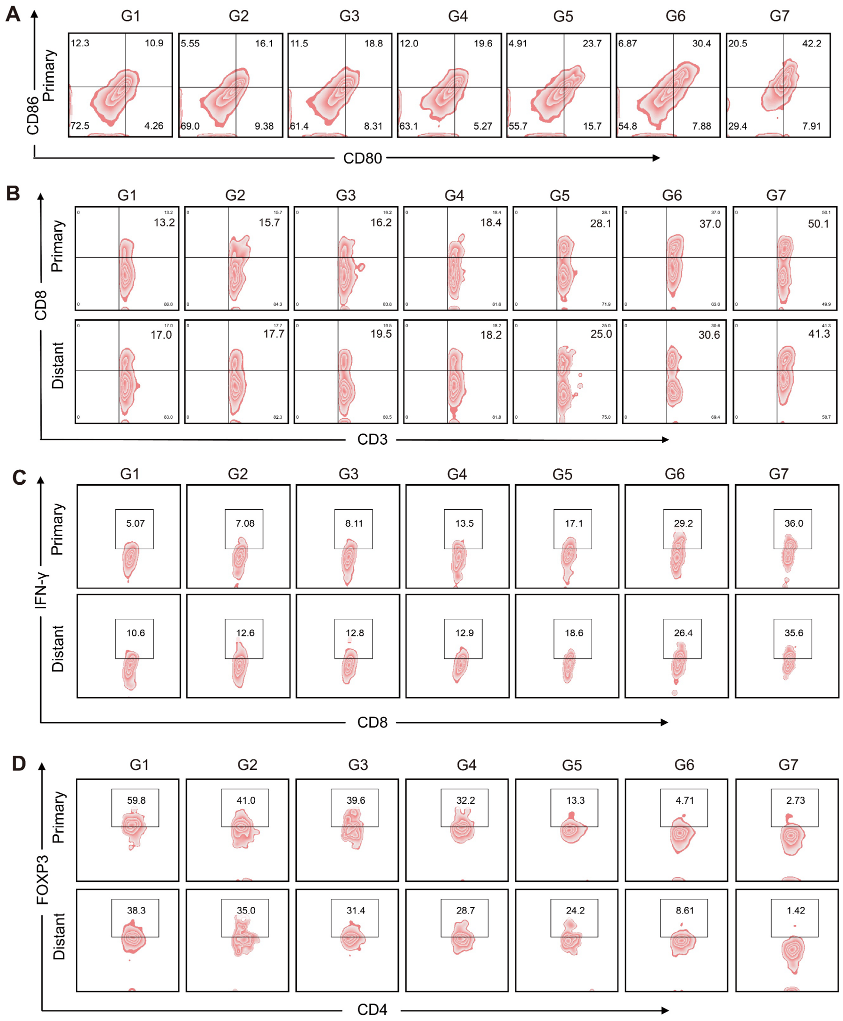

Figure S19. A) The FCM analysis of DCs maturation in tumor-draining lymph nodes adjacent to primary tumors after last treatment for 3 days. Cells are gated by CD11c⁺ cells. The FCM analysis of (B) CD8⁺ T cells (gated by CD3⁺ T cells), (C) CTLs (gated by CD8⁺ T cells), and Tregs (gated by CD4⁺ T cells) (D) in primary and distant tumor tissues after last treatment for 3 days. G1: PBS, G2: β-Lap NPs, G3: αPD-L1, G4: RT, G5: RT + αPD-L1, G6: RT + β-Lap/Fe NPs, G7: RT + β-Lap/Fe NPs + αPD-L1.

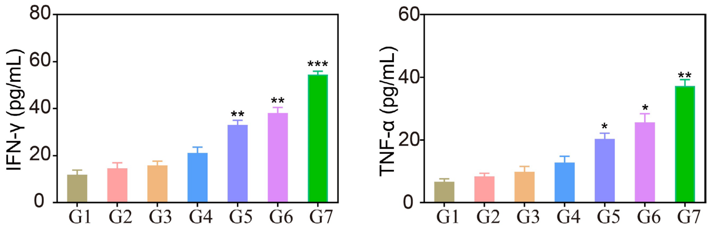

Figure S20. The levels of IFN-γ and TNF-α in bilateral 4T1 tumor-bearing mice serum isolated at last treatment for 3 days. G1: PBS, G2: β-Lap NPs, G3: αPD-L1, G4: RT, G5: RT + αPD-L1, G6: RT + β-Lap/Fe NPs, G7: RT + β-Lap/Fe NPs + αPD-L1. Values are presented as the mean ± SD (n = 3). *P <0.05, **P < 0.01, ***P < 0.001.
